# Loss of Upc2p-Inducible *ERG3* Transcription Is Sufficient To Confer Niche-Specific Azole Resistance without Compromising Candida albicans Pathogenicity

**DOI:** 10.1128/mBio.00225-18

**Published:** 2018-05-22

**Authors:** Arturo Luna-Tapia, Hubertine M. E. Willems, Josie E. Parker, Hélène Tournu, Katherine S. Barker, Andrew T. Nishimoto, P. David Rogers, Steven L. Kelly, Brian M. Peters, Glen E. Palmer

**Affiliations:** a Department of Clinical Pharmacy and Translational Science, College of Pharmacy, University of Tennessee Health Sciences Center, Memphis, Tennessee, USA; b Institute of Life Science, Swansea University Medical School, Swansea, Wales, United Kingdom; Duke University Medical Center

**Keywords:** azole resistance, *Candida albicans*, disseminated candidiasis, *ERG3*, mouse models, pathogenesis, vaginal candidiasis

## Abstract

Inactivation of sterol Δ^5,6^-desaturase (Erg3p) in the prevalent fungal pathogen Candida albicans is one of several mechanisms that can confer resistance to the azole antifungal drugs. However, loss of Erg3p activity is also associated with deficiencies in stress tolerance, invasive hyphal growth, and attenuated virulence in a mouse model of disseminated infection. This may explain why relatively few *erg3*-deficient strains have been reported among azole-resistant clinical isolates. In this study, we examined the consequences of Erg3p inactivation upon C. albicans pathogenicity and azole susceptibility in mouse models of mucosal and disseminated infection. While a C. albicans *erg3*Δ/Δ mutant was unable to cause lethality in the disseminated model, it induced pathology in a mouse model of vaginal infection. The *erg3*Δ/Δ mutant was also more resistant to fluconazole treatment than the wild type in both models of infection. Thus, complete loss of Erg3p activity confers azole resistance but also niche-specific virulence deficiencies. Serendipitously, we discovered that loss of azole-inducible *ERG3* transcription (rather than complete inactivation) is sufficient to confer *in vitro* fluconazole resistance, without compromising C. albicans stress tolerance, hyphal growth, or pathogenicity in either mouse model. It is also sufficient to confer fluconazole resistance in the mouse vaginal model, but not in the disseminated model of infection, and thus confers niche-specific azole resistance without compromising C. albicans pathogenicity at either site. Collectively, these results establish that modulating Erg3p expression or activity can have niche-specific consequences on both C. albicans pathogenicity and azole resistance.

## IMPORTANCE

While conferring resistance to the azole antifungals *in vitro*, loss of sterol Δ^5,6^-desaturase (Erg3p) activity has also been shown to reduce C. albicans pathogenicity. Accordingly, it has been presumed that this mechanism may not be significant in the clinical setting. The results presented here challenge this assumption, revealing a more complex relationship between Erg3p activity, azole resistance, C. albicans pathogenicity, and the specific site of infection. Most importantly, we have shown that even modest changes in *ERG3* transcription are sufficient to confer azole resistance without compromising C. albicans fitness or pathogenicity. Given that previous efforts to assess the importance of *ERG3* as a determinant of clinical azole resistance have focused almost exclusively on detecting null mutants, its role may have been grossly underestimated. On the basis of our results, a more thorough investigation of the contribution of the *ERG3* gene to azole resistance in the clinical setting is warranted.

## INTRODUCTION

Mortality rates associated with invasive fungal infections (IFIs) remain alarmingly high, despite the availability of three major classes of antifungal drugs (1). One of the most prevalent fungal pathogens, Candida albicans, is estimated to cause more than 400,000 IFIs annually, with attributable mortality rates of 35 to 75% (2). *Candida* species are also a leading cause of mucosal disease, including a collection of oral manifestations (3), and vaginal candidiasis, which afflicts 70 to 75% of healthy adult women (4). Aside from episodic infections, 5 to 10% of all women suffer from recurrent vulvovaginal *Candida* infections that cause chronic vaginal pain and require long-term antifungal maintenance therapy to alleviate symptoms (4). Improving patient outcomes for both disseminated and mucosal infections will depend upon a detailed understanding of the causes of treatment failure and the development of countermeasures. The azole antifungals remain the most widely used therapies, acting through inhibition of lanosterol demethylase (Erg11p), an enzyme involved in ergosterol biosynthesis. The resulting depletion of cellular ergosterol and the accumulation of abnormal sterol species, such as 14α-methylergosta-8,24(28)-dien-3β,6α-diol, are both thought to cause plasma membrane dysfunction, ultimately leading to growth arrest (5). Several acquired mechanisms of azole resistance have been characterized in C. albicans, and these mechanisms include overexpression of drug efflux pumps belonging to the major facilitator and ABC transporter superfamilies, as well as the target enzyme itself (6, 7). Mutations that alter the target enzymes’ (Erg11p) azole binding affinity are also important determinants of susceptibility (8–10). However, a combination of the above mechanisms are usually necessary to confer clinically relevant levels of azole resistance (7, 11–13). Notably, the resistance of many fungal isolates is not fully accounted by these mechanisms (14).

An alternative mechanism of azole resistance can arise through inactivation of sterol Δ^5,6^-desaturase (Erg3p), the enzyme responsible for converting 14α-methylfecosterol, which accumulates upon azole-mediated inhibition of Erg11p, into the dysfunctional diol species (5). However, while azole-resistant mutants devoid of Erg3p activity can be readily selected *in vitro* (15), only a handful have been reported among azole-resistant clinical isolates of C. albicans (5, 16–18). This may be because loss of Erg3p activity itself blocks a late step of ergosterol biosynthesis (17), resulting in membrane abnormalities and hypersensitivity to some physiological stresses (19–22). In C. albicans, loss of Erg3p activity has also been associated with defects in hyphal growth (17, 20, 23), a major virulence determinant of this species (24, 25). Accordingly, several reports have described both clinically derived and lab-engineered *erg3* mutants as having attenuated virulence in a mouse model of hematogenously disseminated infection (18, 20, 23). Thus, while inactivation of Erg3p leads to azole resistance, the associated fitness and pathogenicity defects may disfavor the selection of *erg3* mutants in the clinical setting. The significance of *ERG3* inactivating mutations to azole resistance in the clinical setting has been further brought into question by the finding that a C. albicans *erg3Δ/Δ* mutant is susceptible to azole treatment in a mouse model of disseminated infection, despite *in vitro* resistance (23). Either way, to date, the consequences of Erg3p inactivation upon C. albicans pathogenicity and azole resistance at mucosal body sites has not been directly tested. This is important, as we previously reported that C. albicans *erg2Δ/Δ* and *erg24Δ/Δ* ergosterol biosynthetic mutants (lacking C-8 sterol isomerase and C-14 sterol reductase, respectively), are essentially avirulent in the disseminated model but readily colonize the mouse vagina (26).

The purpose of this study was to determine how loss of Erg3p activity affects C. albicans pathogenicity and azole resistance in a mouse model of vaginal candidiasis. In the course of conducting these studies, we serendipitously discovered that altering the regulation of *ERG3* transcription is sufficient to induce *in vitro* and niche-specific *in vivo* azole resistance, without compromising the pathogenic fitness of the fungus.

## RESULTS

### Altering *ERG3* transcription is sufficient to induce *in vitro* azole resistance without compromising Candida albicans fitness.

To determine whether the loss of Erg3p activity affects C. albicans pathogenicity and azole resistance in a murine model of vaginal candidiasis, we first constructed a C. albicans *erg3Δ/Δ* deletion strain using a PCR-based approach (27). A reconstituted strain was then made by reintroducing a wild-type *ERG3* allele, including 582 bp of 5′ untranslated region (5′ UTR) and 303 bp of 3′ UTR into the deletion strain using an integrating vector that fully restores the *IRO1-URA3* locus (28) to circumvent the well-documented positional effects of *URA3* integration (29). A prototrophic (and isogenic) deletion strain was produced by introducing the vector alone into the *erg3Δ/Δ* mutant.

**FIG 1:**
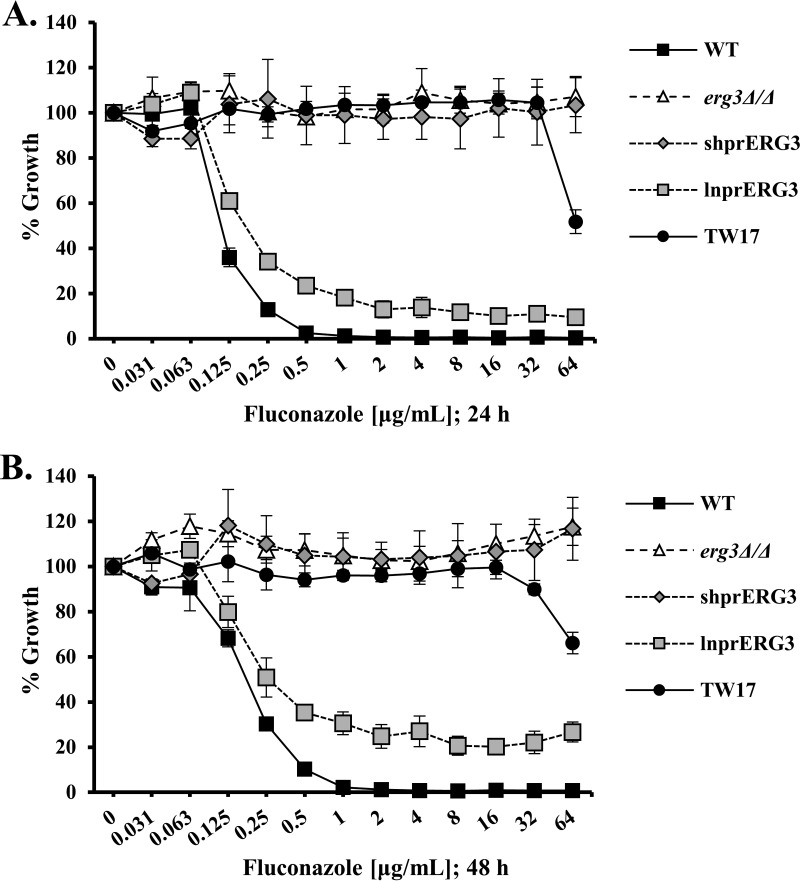
The 5′ UTR of *ERG3* is an important determinant of C. albicans azole susceptibility. The C. albicans *erg3*Δ/Δ mutant was complemented by reintroduction of a wild-type copy of the *ERG3* ORF with either 582 nucleotides (nt) (strain shprERG3) or 1,000 nt (strain lnprERG3) of the 5′ UTR and 303 nt of the 3′ UTR. The fluconazole susceptibility of the wild-type (WT), *erg3*Δ/Δ, shprERG3, and lnprERG3 strains and an azole-resistant clinical isolate (TW17) was evaluated using the standard CLSI broth microdilution protocol. Following 24 h (A) or 48 h (B) of incubation, growth was measured as OD_600 _and expressed as a percentage of the growth in the control wells with no drug (DMSO alone) for each strain. The means ± standard deviations (error bars) for three biological replicates are indicated.

**FIG 2:**
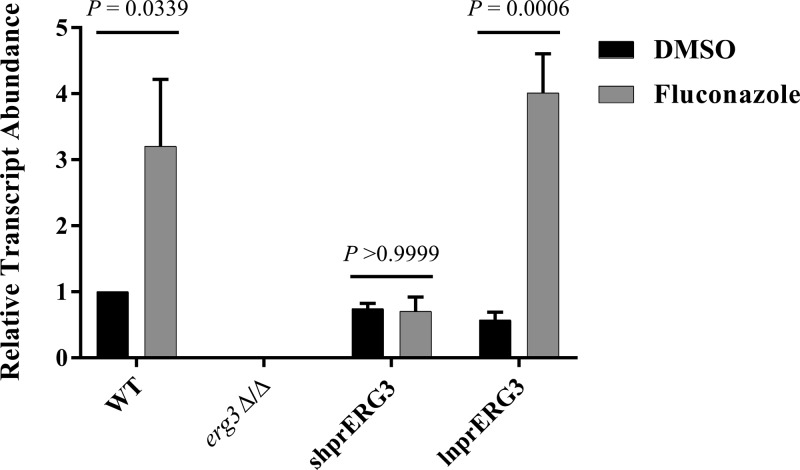
Azole-inducible *ERG3* transcription is lost in the shprERG3 strain. Wild-type (WT), *erg3*Δ/Δ, shprERG3, and lnprERG3 strains were grown for 24 h in YPD at 37°C in the presence or absence of 5 µg/ml fluconazole. RNA was then extracted, *ERG3* transcript abundance was quantified by qRT-PCR using the Δ*C*_*T*_ method, and *ERG3* transcript levels were normalized to that of *ACT1*. The mean plus standard error of the mean (error bar) for three biological replicates are shown for each strain. Each treatment group was compared using a two-way analysis of variance (ANOVA) and Tukey’s multiple-comparison test, and the *P* values are indicated.

To confirm the expected *in vitro* azole resistance of the *erg3Δ/Δ* strain, we tested its susceptibility to fluconazole using the standard CLSI broth microdilution protocol (30). As anticipated, the *erg3Δ/Δ* mutant was insensitive to fluconazole (Fig. 1). Unexpectedly, the *ERG3* reconstituted strain (from here on referred to as shprERG3 [shpr stands for shorter promoter]), was also fully resistant to fluconazole (Fig. 1), voriconazole (see Fig. S1A in the supplemental material), itraconazole (Fig. S1B), and ketoconazole (Fig. S1C). This result was confirmed by testing multiple independently constructed strains of the same genotype. Sequence analysis confirmed the absence of mutations within the cloned *ERG3* sequences. Subsequently, a second *ERG3* reconstituted strain (from here on referred to as lnprERG3 [lnpr stands for longer promoter]) was produced by reintroducing *ERG3* with 1,000 bp of the 5′ UTR, which was sufficient to restore fluconazole susceptibility (Fig. 1). These results initially suggested that insufficient 5′ UTR had been reintroduced into the shprERG3 strain and that some promoter element essential for *ERG3* transcription lies between 1,000 and 582 bp upstream of the start codon. Surprisingly, this was not supported by quantitative reverse transcription-PCR (qRT-PCR) analysis, which revealed similar levels of the *ERG3* transcript in wild-type, shprERG3, and lnprERG3 strains (Fig. 2). However, while the addition of 5 µg/ml fluconazole increased the *ERG3* transcript three- to fourfold in both wild-type and lnprERG3 strains, this response was not observed in the shprERG3 strain. As expected, no *ERG3* transcript was detected in the *erg3Δ/Δ* strain.

10.1128/mBio.00225-18.1 FIG S1  C. albicans *erg3*Δ/Δ and shprERG3 strains are resistant to several azole antifungals. The susceptibility of wild-type (WT) (GP1), *erg3*Δ/Δ, shprERG3, lnprERG3, and an azole-resistant clinical isolate (TW17) to the triazole drugs voriconazole (A) and itraconazole (B) and the imidazole ketoconazole (C) were evaluated using the standard CLSI broth microdilution protocol. Following 48-h incubation, growth was measured as OD_600_ and expressed as a percentage of the growth in the no-drug control wells (DMSO alone) for each strain. Values are means ± standard deviations for three biological replicates. Download FIG S1, JPG file, 1.8 MB. Copyright © 2018 Luna-Tapia et al. 2018 Luna-Tapia et al. This content is distributed under the terms of the Creative Commons Attribution 4.0 International license.

**FIG 3:**
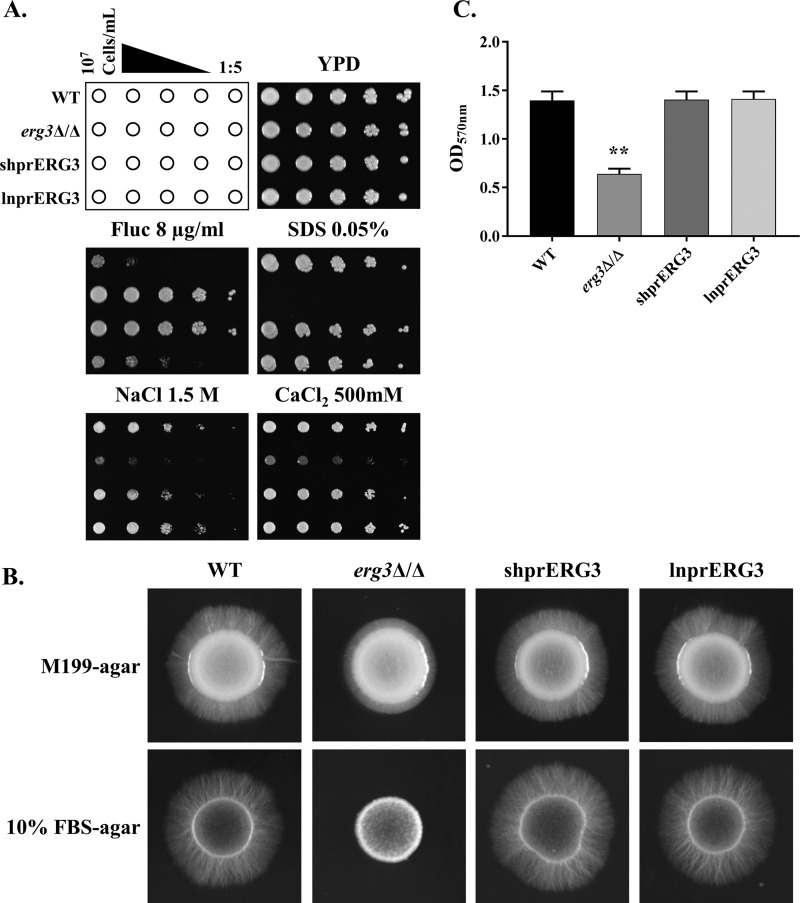
Loss of azole-inducible *ERG3* transcription does not affect C. albicans stress tolerance or hyphal growth. (A) C. albicans cell suspensions were prepared at 1 × 10^7^ cells/ml, and serial 1:5 dilutions prepared in sterile water. Cell suspensions were then applied to YPD agar plates or YPD agar supplemented with the indicated concentrations of fluconazole (Fluc), SDS, NaCl, or CaCl_2 _using a sterile multipronged applicator. The plates were incubated at 30°C for 48 h and then imaged. (B) C. albicans strains were suspended at 1 × 10^7^ cells/ml in sterile water, and 2.5 µl spotted onto either an M199 or 10% FBS agar plate. The resulting colonies were imaged after 96-h incubation at 37°C. (C) The capacity to form biofilms was also evaluated in flat-bottomed 96-well plates. Each C. albicans strain was grown in RPMI 1640 medium at 37°C for 24 h and stained with crystal violet before biofilm mass was quantified as OD_570_. Values are means plus standard errors of the means (SEM) from four independent experiments. The biofilm mass of each group was compared using one-way ANOVA and Tukey’s multiple-comparison test. The value for the *erg3*Δ/Δ mutant group was significantly different (*P* < 0.0001) from the values for the WT, shprERG3, and lnprERG3 groups as indicated by the two asterisks.

Given that the shprERG3 strain expresses apparently normal levels of the *ERG3* transcript in the absence of the azoles, we next examined several other phenotypes that have been associated with Erg3p deficiency (22, 31). The *erg3Δ/Δ* mutant was hypersensitive to membrane stress caused by the detergent SDS, as well as ionic stresses induced by high concentrations of CaCl_2_ or NaCl (Fig. 3A). However, these phenotypes were not observed in either the shprERG3 or lnprERG3 strains, which behaved as the wild-type control.

Several C. albicans Erg3p-deficient strains have also been described as having defects in hyphal growth (17, 18, 20, 23), which is essential for pathogenicity (24, 25). Similarly, we found our *erg3*Δ/Δ mutant to be deficient in hyphal growth on medium 199 (M199) and 10% fetal bovine serum (FBS) agar (Fig. 3B). Under these conditions, both the shprERG3 and lnprERG3 strains formed apparently normal hyphae. Notably, the C. albicans *erg3*Δ/Δ mutant can form hyphae when grown in liquid M199 medium at 37°C (Fig. S2) or when Ume6p, a transcription factor that activates the expression of hypha-specific genes, is overexpressed (Fig. S3). Thus, the C. albicans *erg3*Δ/Δ mutant is capable of forming hyphae under at least some conditions. As hyphal growth is intimately linked to the ability of C. albicans to form biofilms (32), we compared each strain’s capacity to produce biofilms using a simple *in vitro* assay. While the *erg3Δ/Δ* mutant has diminished capacity to form biofilms, both shprERG3 and lnprERG3 strains were unaffected (Fig. 3C).

10.1128/mBio.00225-18.2 FIG S2  C. albicans *erg3*Δ/Δ mutant forms hyphae in liquid M199 medium. Wild-type (WT), *erg3*Δ/Δ, shprERG3, and lnprERG3 strains of C. albicans were subcultured into M199 medium at approximately 1 × 10^6^ cells/ml and incubated at 37°C with shaking. Samples were taken at 4-h intervals and fixed with formalin. Cell morphologies were observed under light microscopy using a 40× objective. Bar = 50 µM. Download FIG S2, JPG file, 1.4 MB. Copyright © 2018 Luna-Tapia et al. 2018 Luna-Tapia et al. This content is distributed under the terms of the Creative Commons Attribution 4.0 International license.

10.1128/mBio.00225-18.3 FIG S3  Overexpression of *UME6* causes constitutive hyphal growth in the C. albicans *erg3*Δ/Δ mutant. The WT CAI4 strain and the *erg3*Δ/Δ mutant were transformed with pKE4-UME6 to overexpress the Ume6p transcription factor or with pKE4 vector alone. The resulting strains were grown in YPD broth overnight at 30°C and observed by light microscopy using a 40× objective. Bar = 100 µM. Download FIG S3, JPG file, 1.8 MB. Copyright © 2018 Luna-Tapia et al. 2018 Luna-Tapia et al. This content is distributed under the terms of the Creative Commons Attribution 4.0 International license.

In summary, reintroducing *ERG3* with the shorter promoter into the *erg3Δ/Δ* strain is sufficient to restore normal stress tolerance and hyphal growth but insufficient to confer *in vitro* fluconazole susceptibility. The phenotypes of the shprERG3 strain demonstrate that reducing *ERG3* transcription or eliminating its azole-inducible expression (rather than complete loss of Erg3p function) is sufficient to dramatically enhance C. albicans survival following azole exposure, and without compromising fungal fitness or virulence-related attributes.

### Azole-induced *ERG3* transcription is dependent upon the Upc2p transcription factor.

To provide a convenient reporter of transcriptional activity, either 582 bp (short promoter) or 1,000 bp (long promoter) of the *ERG3* 5′ UTR was amplified from strain SC5314 and cloned upstream of the green fluorescent protein gamma variant (GFPγ) coding sequence (33). Each reporter construct was then introduced into the wild-type strain CAI4 (34). Consistent with the qRT-PCR data, in the absence of fluconazole, GFPγ expression was similar for either construct. While fluconazole treatment increased the GFPγ signal approximately eightfold in the strain carrying the longer promoter, no increase was observed in the strain with the shorter *ERG3* promoter (Fig. S4). This further supports the existence of a promoter element, located between 582 and 1,000 bp upstream of the *ERG3* open reading frame (ORF) that is required to activate transcription in response to azole treatment. To determine whether *ERG3* transcription may be affected by changes in growth conditions, we examined the effects of temperature (30°C, 35°C, and 37°C) as well as pH (pH 3, 4, 5, 6, and 7) using the *P*_*ERG3*_-GFPγ reporter constructs. However, neither parameter had a significant impact on GFPγ expression from either the long or short promoters (less than twofold, data not shown).

10.1128/mBio.00225-18.4 FIG S4  Truncation of the 5′ UTR of *ERG3* results in loss of azole-inducible transcription. Reporter constructs consisting of 1,000 bp (*P*_*lnERG3*_) or 582 bp (*P*_*shERG3*_) of the *ERG3* 5′ UTR from strain SC5314 placed upstream of the GFPγ coding sequence were introduced into C. albicans strain CAI4 (WT). The resulting strains were grown in YNB medium with or without 5 µg/ml fluconazole for 24 h at 35°C with shaking. GFPγ fluorescence intensity was quantified (excitation wavelength of 488 nm; emission wavelength of 507 nm), normalized for cell density (OD_600_), and expressed relative to that of the DMSO control of the *P*_*lnERG3*_ reporter. Values are means ± standard deviations for four biological replicates. The groups were compared by using a two-way ANOVA and Tukey’s multiple-comparison test. Download FIG S4, JPG file, 0.5 MB. Copyright © 2018 Luna-Tapia et al. 2018 Luna-Tapia et al. This content is distributed under the terms of the Creative Commons Attribution 4.0 International license.

**FIG 4:**
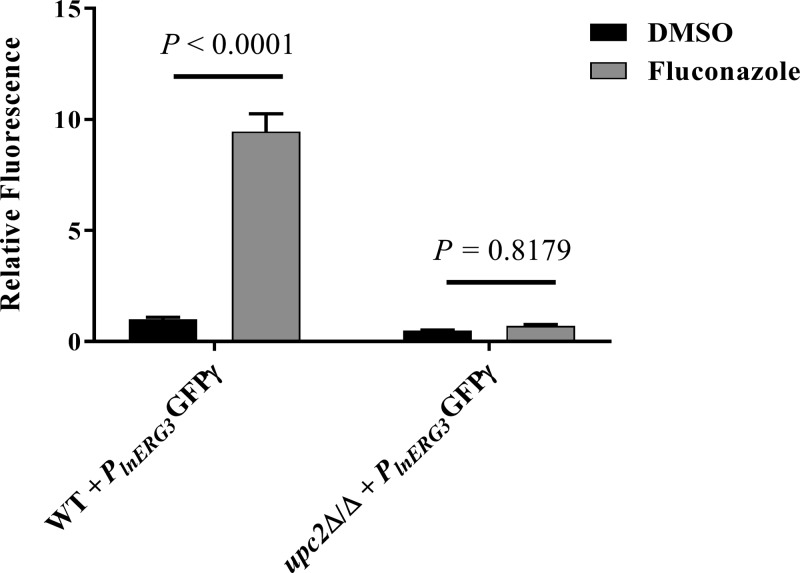
Azole-inducible *ERG3* transcription is dependent upon the Upc2p transcription factor. The GFPγ coding sequence was cloned downstream of 1 kb of the *ERG3* promoter sequence, and the resulting construct introduced into C. albicans wild-type strain CAI4 (WT) or a *upc2*Δ/Δ mutant. The resulting strains were grown in YNB broth in the presence or absence of 5 µg/ml fluconazole for 24 h at 35°C with shaking, before GFPγ fluorescence was quantified (excitation wavelength of 488 nm [9 nm bandpass]; emission wavelength of 507 nm [9 nm bandpass]). Fluorescence intensity was then normalized to the cell density (OD_600_) and expressed relative to the DMSO control. The means ± the standard deviations for four biological replicates are shown. The relative fluorescence of each treatment group was then compared using a two-way ANOVA and Tukey’s multiple-comparison test, and the *P* values are indicated.

In C. albicans, the Upc2p transcription factor enhances the expression of several ergosterol biosynthetic genes in response to azole treatment, including *ERG11* (35). Although *ERG3* appears to be a target of Upc2p in Saccharomyces cerevisiae (36), this had not been confirmed in C. albicans. To address this question, we introduced the azole-responsive GFPγ reporter construct (with the longer promoter) into an *upc2*Δ/Δ deletion strain. In the absence of fluconazole, GFPγ expression was similar in the *upc2*Δ/Δ mutant and wild-type strain backgrounds, indicating similar levels of basal transcription (Fig. 4). Fluconazole treatment increased GFPγ expression about 10-fold in the wild-type strain, but not in the *upc2*Δ/Δ mutant. These results confirm that the induction of *ERG3* transcription that occurs in C. albicans upon azole treatment is completely dependent upon the Upc2p transcription factor.

Using the program matrix-scan at the Regulatory Sequence Analysis Tool RSAT-Fungi webserver (http://rsat-tagc.univ-mrs.fr/rsat/matrix-scan_form.cgi) (37), we searched for motifs that may represent putative Upc2p-binding sites within the 5′ UTR of *ERG3*. A *cis*-regulatory element-enriched region (CRER) was identified between positions −751 to −373. Within this region, four motifs represent putative Upc2p-binding sites (Fig. S5). Another element located at positions −379 to −373 (TCGTTTA) that coincides with a putative Upc2-binding site resembles the classical sterol-responsive element (SRE) TCGTATA, present in the 5′ UTRs of *UPC2* and several ergosterol biosynthetic genes (38–40); hence, we deemed this sequence an “imperfect” SRE (iSRE) (Fig. S5). The *ERG3* promoter also contains two elements that match the TATA box consensus sequence TATAWAWR (W means A or T, and R means A or G) (41); the first element is located at positions −679 to −672, and the second element is located at positions −343 to −336 (Fig. S5). Nonetheless, the role of each of the above elements in *ERG3*’s transcriptional upregulation in response to the azoles has not yet been determined.

10.1128/mBio.00225-18.5 FIG S5  Multiple regulatory elements are located within 1 kb of the C. albicans *ERG3* 5′ UTR. Motifs that represent putative Upc2p-binding sites (orange bars) were identified using the program matrix-scan at the Regulatory Sequence Analysis Tool RSAT-Fungi webserver (http://rsat-tagc.univ-mrs.fr/rsat/matrix-scan_form.cgi). The scan was performed using the JASPAR core fungi collections 2018 and the matrix identifier UPC2 - MA0411.1 in transfac format. A Markov order 1 and C. albicans genome were used for the background model estimation method. Scanning was performed on both strands, with a *cis*-regulatory element-enriched region (CRER) size of 0 to 500 bp, and the default upper detection threshold *P* value (0.001). Putative TATA boxes (light blue bars) were identified using the DNA-pattern tool at the RSAT-fungi server and the TATA box consensus sequence TATAWAWR (W is A/T; R is A/G) as the query. Segments of the *ERG3* 5′ UTR that correspond to the “short” (582-bp) and “long” (1-kb) promoter variants are indicated in gray or dark blue lines, respectively. DRE, direct repeat element (unknown function). Download FIG S5, JPG file, 1.6 MB. Copyright © 2018 Luna-Tapia et al. 2018 Luna-Tapia et al. This content is distributed under the terms of the Creative Commons Attribution 4.0 International license.

### Loss of azole-inducible *ERG3* transcription is sufficient to eliminate “toxic” diol production in fluconazole-treated C. albicans.

In order to compare levels of Erg3p activity, we analyzed the composition of sterols extracted from both untreated and fluconazole-treated cultures of each strain. As expected, untreated wild-type and lnprERG3 cells produced an abundance of ergosterol (Table 1). In contrast, the *erg3Δ/Δ* mutant did not produce detectable levels of ergosterol under untreated conditions, instead accumulating high levels of ergosta-7,22-dienol. The addition of fluconazole reduced ergosterol content in the wild-type and lnprERG3 strains by more than 25-fold and induced the formation of significant levels of 14α-methylergosta-8,24(28)-dien-3β,6α-diol (Table 2). However, *erg3Δ/Δ* cells did not form the dysfunctional diol species upon fluconazole treatment. Interestingly, while the shprERG3 strain formed near normal levels of ergosterol in the absence of drug (Table 1), it failed to produce detectable levels of the sterol-diol species upon fluconazole treatment (Table 2). The capacity to produce ergosterol in the absence of drug explains the lack of stress and hyphal growth phenotypes for the shprERG3 strain. The azole resistance of this strain can also be explained by the absence of abnormal diols upon antifungal treatment.

**TABLE 1:** Sterol profiles of untreated C. albicans *erg3*Δ/Δ mutant and control strains

Sterol	Sterol content^a^ (%) (mean ± SD) in strain:			
	WT	*erg3*Δ/Δ	shprERG3	lnprERG3
Squalene	8.9 ± 0.6	3.0 ± 0.3	6.5 ± 0.5	7.7 ± 1.0
Unknown sterol^b^	ND	0.2 ± 0.0	ND	ND
Ergosta-8,22-dienol	ND	8.0 ± 0.4	ND	ND
Ergosta-8,22,24-trienol	ND	0.8 ± 0.0	ND	ND
Ergosta-5,8,22,24(28)-tetraenol	**1.3** ± 0.1	ND	**0.8** ± 0.0	**1.2** ± 0.0
Ergosta-5,8,22-trienol	**0.6** ± 0.0	ND	**0.3** ± 0.0	**0.6** ± 0.1
Zymosterol	8.0 ± 0.2	ND	5.1 ± 0.4	5.7 ± 0.5
Ergosterol	**58.4** ± 0.7	ND	**43.1** ± 0.5	**58.4** ± 2.8
Ergosta-7,22-dienol	ND	35.9 ± 0.5	5.8 ± 0.5	1.3 ± 0.4
Ergosta-5,7,22,24-tetraenol	**1.8** ± 0.1	ND	**0.9** ± 0.1	**1.2** ± 0.1
Ergosta-7,22,24(28)-trienol	ND	0.5 ± 0.1	ND	ND
Fecosterol [E8,24(28)]	4.5 ± 0.1	16.8 ± 1.4	10.4 ± 0.3	5.3 ± 0.3
Ergosta-8-enol	ND	2.7 ± 1.8	1.0 ± 0.0	0.5 ± 0.1
14-Methylfecosterol	ND	0.5 ± 0.1	ND	ND
Ergosta-5,7,24(28)-trienol	**1.3** ± 1.0	ND	**1.2** ± 0.5	**2.2** ± 0.8
4-Methylzymosterol	ND	0.4 ± 0.0	ND	ND
Ergosta-5,7-dienol	**4.2** ± 0.2	ND	**2.1** ± 0.3	**3.0** ± 0.3
Episterol	5.1 ± 0.1	24.1 ± 1.4	18.0 ± 0.3	8.9 ± 0.4
Ergosta-7-enol	0.2 ± 0.1	3.6 ± 0.3	0.9 ± 0.2	0.4 ± 0.0
Lanosterol	4.1 ± 0.3	2.0 ± 0.2	2.7 ± 0.3	2.7 ± 0.2
4-Methylfecosterol	ND	0.3 ± 0.0	ND	ND
4,4-Dimethylcholesta-8,24-dienol	1.4 ± 0.0	0.5 ± 0.1	0.7 ± 0.0	0.8 ± 0.1
Unknown sterol^b^	ND	0.4 ± 0.1	ND	ND
Eburicol	0.2 ± 0.0	0.3 ± 0.0	0.2 ± 0.0	0.2 ± 0.0

Σ(“*ERG3* sterols”)	**67.6** ± 2.1	**0.0** ± 0.0	**48.6** ± 1.4	**66.6** ± 4.1

a The content of sterols indicative of Erg3p activity is shown in boldface type. ND, not detected.

b Sterols that have not been identified (the mass ion and/or fragmentation pattern was unclear).

**TABLE 2:** Sterol profiles of fluconazole-treated C. albicans *erg3*Δ/Δ mutant and control strains

Sterol	Sterol content^a^ (%) (mean ± SD) in strain:			
	WT	*erg3*Δ/Δ	shprERG3	lnprERG3
Ergosterol	**2.0** ± 0.2	ND	**0.7** ± 0.1	**1.0** ± 0.6
Ergosta-7,22-dienol	ND	1.3 ± 0.1	0.5 ± 0.0	ND
4,14-Dimethylzymosterol	11.3 ± 0.3	14.7 ± 1.0	14.3 ± 0.6	12.1 ± 0.8
14-Methylfecosterol	4.5 ± 0.2	1.7 ± 0.2	2.3 ± 0.1	2.5 ± 0.1
4,4-Dimethyl-ergosta-8,14,24(28)-trienol	1.2 ± 0.1	0.8 ± 0.0	0.7 ± 0.1	0.8 ± 0.0
14-Methyl-ergosta-8,24(28)-dien-3-6-diol	**2.7** ± 0.5	ND	ND	**1.1** ± 0.4
Lanosterol	41.6 ± 0.3	33.0 ± 0.6	33.6 ± 0.9	36.0 ± 0.3
14-Methyl-ergosta-trienol^b^	1.1 ± 0.1	1.0 ± 0.1	1.0 ± 0.0	0.9 ± 0.1
Eburicol	35.5 ± 0.7	47.6 ± 1.7	47.0 ± 1.7	45.6 ± 0.8

Σ(“*ERG3* sterols”)	**4.7** ± 0.7	**0.0** ± 0.0	**0.7** ± 0.1	**2.2** ± 0.9

a The content of sterols indicative of Erg3p activity is shown in boldface text. ND, not detected.

b In some cases (e.g., 14-methyl-ergosta-trienol), we cannot be certain of the arrangement of double bonds in the sterol, but it is an ergosta type sterol with three double bonds and a 14-methyl group.

### Loss of azole-inducible *ERG3* transcription does not compromise C. albicans pathogenicity, but it is insufficient to confer fluconazole resistance in a mouse model of disseminated infection.

**FIG 5:**
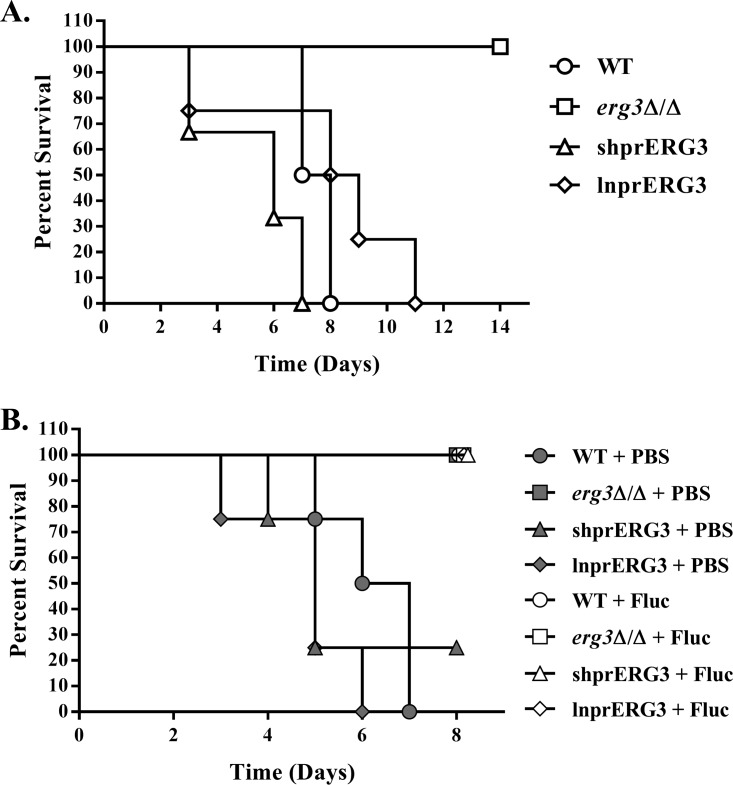
The shprERG3 strain is virulent but susceptible to fluconazole treatment in a mouse model of disseminated candidiasis. (A) BALB/c mice (six mice in each group) were inoculated with ~5 × 10^5^ CFU of either the WT (GP1), *erg3*Δ/Δ, shprERG3, or lnprERG3 strain via lateral tail vein injection. The mice were then monitored three times daily for 14 days, and those showing distress were humanely euthanized. The survival of each group was compared using the log rank test (*P* = 0.0051). (B) Groups of BALB/c mice were inoculated with ~5 × 10^5^ CFU of either WT (GP1), *erg3*Δ/Δ, shprERG3, or lnprERG3 strain as described above and then split into two treatment groups (four mice in each group). One group was treated with 25 mg/kg fluconazole (Fluc), and another group was treated with vehicle alone (PBS) by daily intraperitoneal injection starting 24 h after infection. Animals were monitored for 8 days, and the survival of each group was compared using the log rank test (*P* < 0.0001).

It has been previously reported that deletion of *ERG3* in the SC5314 strain background reduces C. albicans virulence in the mouse model of disseminated candidiasis (20, 21, 23). We therefore compared the pathogenicity of our *erg3Δ/Δ*, shprERG3, and lnprERG3 strains to a wild-type control using the same model. This confirmed that our *erg3Δ/Δ* strain is severely impaired in this model, as it failed to cause mortality within 14 days of inoculation (Fig. 5A). In contrast, the virulence of both the shprERG3 and lnprERG3 strains was indistinguishable from that of the wild-type strain, with each causing 100% mortality. This further supports our earlier conclusion that the fitness and pathogenicity of the shprERG3 strain are not compromised, despite its *in vitro* azole resistance. Interestingly, while the *erg3Δ/Δ* deletion strain did not cause mortality, it was able to persist within the mouse tissues to the end of the experiment {(~2.03 ± 1.32) × 10^6^ CFU/g [mean ± standard error of the mean (SEM)]; day 14 postinoculation}. However, the *erg3*Δ/Δ mutant showed defects in hypha formation in this model of infection (Fig. S6).

10.1128/mBio.00225-18.6 FIG S6  The C. albicans *erg3*Δ/Δ mutant shows defects in hyphal formation during disseminated infection. Kidney homogenates from animals infected by tail vein injection with the wild-type (WT), *erg3*Δ/Δ, shprERG3, or lnprERG3 strain of C. albicans were treated with 20% KOH, and visualized by light microscopy using a 40× objective. Bar = 50 µM. Download FIG S6, JPG file, 2 MB. Copyright © 2018 Luna-Tapia et al. 2018 Luna-Tapia et al. This content is distributed under the terms of the Creative Commons Attribution 4.0 International license.

To compare the *in vivo* susceptibility of these strains to fluconazole, a second experiment was conducted in which groups of mice (four mice in each group) were inoculated intravenously with each strain and then either treated for 7 days with fluconazole (25 mg/kg of body weight) or sham treated with phosphate-buffered saline (PBS) via daily intraperitoneal injections. Consistent with the previous experiment, nearly all mice infected with the wild type, shprERG3, and lnprERG3 and treated with PBS succumbed to infection, whereas those mice treated with fluconazole all survived to day 8 (Fig. 5B). Analysis of fungal colonization of the kidneys revealed that fluconazole-treated mice had only low levels of fungal colonization {(8.77 ± 1.33) × 10^2^ CFU/g [mean ± SEM] for the wild-type, (2.97 ± 1.26) × 10^3^ CFU/g for shprERG3, and (2.64 ± 0.14) × 10^2^ CFU/g for lnprERG3}, indicating that all three strains are susceptible to fluconazole in this model. Thus, while the shprERG3 strain is resistant *in vitro*, it is apparently susceptible to fluconazole in the mouse model of disseminated infection. The *erg3Δ/Δ* deletion strain failed to cause lethality in either the PBS or fluconazole treatment groups. However, kidney CFU counts from mice surviving to day 8 were not significantly different between the groups treated with fluconazole [(3.66 ± 2.34) × 10^4^ CFU/g] and PBS [(2.22 ± 0.49) × 10^5^ CFU/g]. Thus, while the *erg3Δ/Δ* strain is much less virulent than the other three strains in the mouse model of disseminated infection, the persistent levels of CFU in the kidney indicate that it is apparently much more resistant to fluconazole treatment.

### Complete or partial loss of Erg3p activity is sufficient to confer fluconazole resistance in a mouse model of vaginal candidiasis.

**FIG 6:**
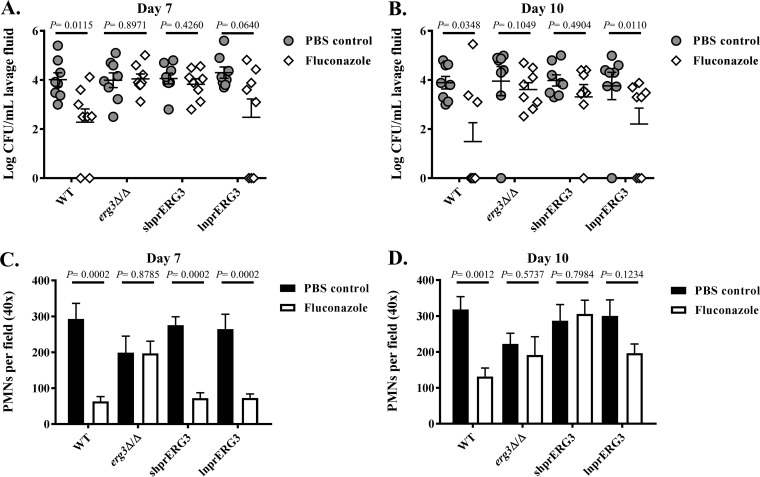
The *erg3*Δ/Δ and shprERG3 C. albicans strains are resistant to fluconazole treatment in a mouse model of vaginal candidiasis. Four groups of 16 estrogen-treated female C57BL/6 mice were each inoculated intravaginally with either the WT (GP1), *erg3*Δ/Δ mutant, shprERG3, or lnprERG3 strain of C. albicans. Each group was then split into two treatment groups (*n* = 8). One group was treated with 50 mg/kg of fluconazole, and the second group was treated with vehicle alone (PBS) via intraperitoneal injection on days 4 and 7 postinfection. The levels of fungal colonization were compared 7 days (A) and 10 days (B) postinfection by quantifying CFU from vaginal lavage fluid. Polymorphonuclear leukocyte (PMN) infiltration into the vaginal mucosa was also compared from smears of lavage fluid taken on day 7 (C) or 10 (D) and stained by the Papanicolau technique. The number of PMNs were then determined by counting the number in five nonadjacent fields using a light microscope with a 40× objective. The mean ± standard error of the mean (SEM) are shown for all panels. Pairwise comparisons between treatment groups were performed using one-way ANOVA analysis with Kruskal-Wallis test.

Previous investigations of Erg3p’s role in C. albicans pathogenicity and azole resistance have focused exclusively on the disseminated model of infection (20, 21, 23). However, it is unknown whether loss of Erg3p activity is sufficient to impair C. albicans virulence or confer azole resistance in other host niches, such as the mucosal membranes that are its primary habitat. To test this, we inoculated estrogen-treated mice intravaginally with either wild-type, *erg3*Δ/Δ, shprERG3, or lnprERG3 strains. Each group was then split into two treatment groups, with half the mice treated with fluconazole (50 mg/kg) via intraperitoneal injection on days 4 and 7 postinoculation, and the other half receiving PBS vehicle alone. The levels of fungal colonization were then determined as CFU from vaginal lavage fluid obtained on days 7 and 10. All four strains were able to colonize the PBS-treated mice to similar levels at both time points (Fig. 6A and B). Fluconazole treatment significantly reduced fungal colonization by both wild-type and lnprERG3 strains but had less impact in animals infected with the *erg3*Δ/Δ or shprERG3 strain (Fig. 6A and B). This indicates that both *erg3*Δ/Δ and shprERG3 strains are not only proficient at colonizing the mouse vagina but also have reduced susceptibility to fluconazole treatment at this site.

Smears from vaginal lavage fluid samples were also made on days 7 and 10 to compare the levels of polymorphonuclear leukocyte (PMN) infiltration, as high levels of PMN influx have been associated with symptomatology (42). Seven days postinoculation, mice infected with the *erg3Δ/Δ* mutant had only a slight reduction in PMN counts compared to the other three strains, further supporting that the mutant has no major virulence defect in this model of infection (Fig. 6C). On day 7, the fluconazole treatment groups had reduced PMN influx in animals infected with the wild-type, shprERG3, and lnprERG3 strains compared to the untreated groups. In contrast, fluconazole had no impact on PMN influx in animals infected with the *erg3Δ/Δ* mutant (Fig. 6C). By day 10, only mice infected with the wild-type strain had a statistically significant reduction in PMN influx in the treated versus untreated groups (Fig. 6D). Similar levels of PMNs were observed in the treated and untreated animals infected with either the *erg3Δ/Δ* or shprERG3 strain, indicating little or no benefit to treatment. For mice infected with the lnprERG3 strain, there was a trend toward reduced PMN influx in the fluconazole-treated group; however, this was not statistically significant. Finally, the hyphae observed in vaginal lavage fluid from mice infected with the *erg3Δ/Δ* mutant were indistinguishable from hyphae formed by the other three strains in this model (Fig. S7). Thus, the *erg3Δ/Δ* mutant is not deficient in hyphal growth at this mucosal site. These findings indicate that the *erg3*Δ/Δ mutant has no colonization defect and is able to induce pathology in the mouse model of vaginal candidiasis. Furthermore, in contrast to the wild type, the capacity of the *erg3*Δ/Δ mutant to induce pathology is not compromised by fluconazole treatment.

10.1128/mBio.00225-18.7 FIG S7  The C. albicans *erg3*Δ/Δ mutant forms hyphae in the vaginal mucosa. Vaginal lavages were performed on mice 10 days after inoculation with wild-type (WT), *erg3*Δ/Δ, shprERG3, and lnprERG3 strains of C. albicans. Following treatment with 20% KOH, each strain was observed by light microscopy using a 40× objective. Download FIG S7, JPG file, 2.2 MB. Copyright © 2018 Luna-Tapia et al. 2018 Luna-Tapia et al. This content is distributed under the terms of the Creative Commons Attribution 4.0 International license.

## DISCUSSION

*In vitro* selection studies with several fungal species have demonstrated that azole resistance frequently results from mutations that inactivate the sterol C-5-desaturase encoded by *ERG3* (15, 43, 44). However, relatively few Erg3p-deficient mutants have been identified among azole-resistant clinical isolates of C. albicans (5, 16–18), which instead seem to favor mechanisms involving alterations of the target protein’s amino acid sequence, increased target protein expression, and/or elevated expression of drug efflux pumps (6–10). This is somewhat surprising, as the acquisition of clinically relevant levels of azole resistance usually requires some combination of these mechanisms, and is thought to be a stepwise process (7, 11–13). In contrast, inactivation of Erg3p can offer a single step mechanism to complete azole resistance. Several factors may help explain the apparent disparity between the high frequency with which azole-resistant *erg3* mutants can be selected *in vitro* and their infrequent occurrence in the clinical setting.

First, the ploidy of the fungal species in question is likely to affect the frequency with which *erg3* mutants arise, as *erg3* loss-of-function mutations are recessive (15). As such, while azole-resistant *erg3* mutants can be readily selected in a haploid species such as S. cerevisiae (15, 44–46), they are expected to occur less frequently in a constitutively diploid species such as C. albicans, as inactivation of both *ERG3* alleles is necessary to increase MICs. This would require either two independently occurring mutations in either allele, or mutation in one allele followed by a gene conversion event. In contrast, activating mutations in the Upc2p transcription factor, which controls expression of the target enzyme Erg11p, or those controlling expression of the *Candida* drug resistance (CDR)- and multidrug resistance (MDR)-type drug efflux pumps (Tac1p and Mrr1p), can be either semidominant or fully dominant (13, 47–49). Nonetheless, *in vitro* selection of Candida dubliniensis, a diploid species, with itraconazole primarily selects for Erg3p-deficient resistant mutants (43). Thus, ploidy alone does not satisfactorily account for the low frequency with which azole-resistant *erg3* mutants are isolated in the clinical setting.

A second factor may be the concentration of drug to which the fungus is exposed. Anderson et al. reported that *in vitro* exposure of S. cerevisiae isolates to high doses of fluconazole primarily selects for *erg3* mutants, while progressive selection with stepwise increases in fluconazole concentration favors resistance mechanisms that involve elevated expression of drug efflux pumps (15). As such, it is conceivable that patient dosing and drug availability at the site of infection are important determinants of the adaptive mechanisms that arise.

A third, possible explanation for the infrequent isolation of *erg3* mutants in the clinical setting may be the associated fitness costs. It is clear from our data, as well as several other studies, that while complete loss of Erg3p activity leads to *in vitro* azole resistance, it concurrently causes several phenotypic abnormalities. These abnormalities include hypersensitivity to various physiological stresses, as well as deficiencies in hyphal growth under at least some culture conditions. The resulting loss of pathogenic fitness may oppose or disfavor the selection of *erg3* mutants in patients receiving azole treatment. While the alternative sterol species that accumulate in *erg3* null mutants are sufficient to support propagation in lab culture, they may be less compatible with fungal survival, proliferation, or invasiveness during infection of the mammalian host. This is supported by several studies that have shown that *erg3* mutants isolated from the clinical setting and lab-engineered *erg3Δ/Δ* strains are less virulent than wild-type isolates in murine models of disseminated infection (18, 20, 23). Interestingly, Vale-Silva et al. reported an azole-resistant C. albicans clinical isolate with an *erg3* null mutation, which is apparently virulent, and had no obvious defects in stress tolerance or hyphal growth (17). The authors suggest that this isolate may harbor compensatory mutations that counteract the effects of Erg3p inactivation. The nature of the compensatory mechanism(s) and the frequency with which they can coincide with *erg3* inactivating mutations are unknown. Nonetheless, the weight of evidence, including the *in vitro* data presented herein, suggests that in the absence of compensatory mechanisms or mutations, complete loss of *ERG3* function is detrimental to C. albicans pathogenic fitness. This notion was only partially supported by our *in vivo* data, which revealed that the *erg3Δ/Δ* mutant has niche-specific defects in pathogenicity.

In both disseminated and vaginal models of infection, wild-type C. albicans is pathogenic but susceptible to azole treatment. In contrast, the *erg3Δ/Δ* mutant has severely attenuated virulence in the mouse model of disseminated candidiasis but is proficient at colonizing and capable of inducing pathology in the vaginal model of infection. Thus, the fitness defects incurred upon Erg3p inactivation are apparently of greater consequence during disseminated infection than mucosal propagation and/or infection. This may reflect the fact that C. albicans is highly adapted for a commensal lifestyle upon mucosal surfaces and typically only disseminates in a highly debilitated mammalian host. Our previous finding that C. albicans *erg2Δ/Δ* and *erg24Δ/Δ* mutants (lacking C-8 sterol isomerase and C-14 sterol reductase, respectively) are avirulent in the mouse model of disseminated infection but readily colonize the mouse vagina (26) further supports the notion that mucosal environments may be more permissive of alterations in the ergosterol biosynthetic pathway than deeper infection sites. Either way, the deletion mutant is relatively resistant to fluconazole treatment in both models of infection. As such, while the *erg3Δ/Δ* mutant has niche-specific virulence defects, it is more azole-resistant than the wild type is at both mucosal and disseminated sites of infection (Table 3).

**TABLE 3:** Summary of pathogenicity and azole susceptibility results in the mouse models of vaginal and disseminated candidiasis

Strain	Summary of results^a^			
	Vaginal infection		Disseminated infection	
	Pathogenicity	Azole resistance	Pathogenicity	Azole resistance
WT	*****	*	*****	*
*erg3*Δ/Δ	*****	*****	**	***
shprERG3	*****	*****	*****	*
lnprERG3	*****	**	*****	*

a Each phenotype is scored on a scale of 1 to 5 with * indicating little or no pathogenicity/azole resistance and ***** indicating full pathogenicity/azole resistance.

Our results with the shprERG3 strain indicate that complete loss of Erg3p activity is not necessary to confer azole resistance. Specifically, the loss of azole-inducible transcription of *ERG3* apparently confers fluconazole resistance *in vitro* without compromising C. albicans pathogenic fitness. Like the wild type, the shprERG3 strain was pathogenic in both models of infection and susceptible to azole treatment in the disseminated model. However, the shprERG3 strain was more resistant to fluconazole treatment in the vaginal model than the wild type, and therefore exhibits niche-specific azole resistance (Table 3). These findings imply that the level of expression and specific activity of Erg3p directly impact C. albicans pathogenicity and susceptibility to azole therapy. Moreover, titrating Erg3p expression/activity has niche-specific consequences upon these vitally important characteristics.

The fourth possible explanation for the dearth of Erg3p-deficient mutants in the clinical setting may be that many are overlooked, as C-5 desaturase activity is not routinely examined. Point mutations and polymorphisms within *ERG3* can be readily detected through sequence analysis, and isolates bearing nonsense mutations—resulting in a truncated (nonfunctional) polypeptide are easily identified. However, the consequences of missense mutations or polymorphisms within the 5′ and 3′ noncoding regions upon Erg3p expression, specific activity, or azole susceptibility are rarely investigated. A detailed biochemical analysis of cellular sterol content is required to confirm deficiencies in C-5 desaturase activity—an analysis that is not routinely performed on all azole-resistant isolates. If this analysis is performed, isolates with a substantive reduction in C-5 sterol desaturase activity can be detected as having significantly reduced ergosterol content (or its complete absence) and an accumulation of ergosta-7,22-dienol. Accordingly, to date, most reports have focused upon describing clinical isolates with mutations that completely inactivate the *ERG3* gene. However, isolates with only a partial reduction in Erg3p activity will have more subtle shifts in sterol profiles that may not be as obvious. This is important, as the results described herein suggest relatively small adjustments to *ERG3* transcription may be sufficient to induce azole resistance in C. albicans—at least within some host niches. The Kelly lab has previously shown that many of the azole-resistant S. cerevisiae *erg3* mutants they selected *in vitro* are in fact “leaky” mutants that only partially impair Erg3p catalytic activity (45). Through thorough analysis of sterol profiles, at least two leaky *erg3* mutants were also identified among a panel of azole-resistant clinical C. albicans isolates (16). Such mutations permit the production of at least some ergosterol but are also sufficient to induce full azole resistance *in vitro* (16). Notably, the two leaky *erg3* C. albicans mutants show phenotypic profiles similar to the profile of the shprERG3 strain reported here, i.e., full azole resistance *in vitro* but normal hyphal growth and stress tolerance (see Fig. S7 in the supplemental material). Our findings beg a series of important questions: To what degree does Erg3p activity need to be suppressed to induce azole resistance? To what degree can Erg3p activity be suppressed before the fitness or pathogenicity of C. albicans is compromised? Are there levels of Erg3p activity that can support full azole resistance without compromising C. albicans pathogenicity? Do the Erg3p activity levels required to support C. albicans pathogenicity and/or determine azole susceptibility/resistance vary between different host niches (e.g., oral versus vaginal versus disseminated) or according to host immune status? Our results also raise the question of whether, and how, altered transcriptional regulation might confer Erg3p-dependent clinical azole resistance that may not be apparent *in vitro* using the standard CLSI MIC assay. Similarly, are there host relevant factors that impinge upon the regulation of Erg3p expression or activity? In this regard, using the *P*_*ERG3*_-GFPγ, we found that β-estradiol (1.25 to 10 µg/ml), which was used in the vaginal model, did not affect transcription from the *ERG3* promoter in the presence or absence of fluconazole (not shown). Finally, and perhaps most importantly, given the almost exclusive focus thus far on *erg3* null mutants—might the role of Erg3p as a determinant of C. albicans azole resistance have been grossly underestimated?

In summary, we have demonstrated that relatively minor adjustments in the regulation of *ERG3* transcription are sufficient to induce azole resistance without compromising C. albicans hyphal growth or stress tolerance. Furthermore, even complete loss of Erg3p activity does not compromise C. albicans pathogenic fitness in a murine model of vaginal candidiasis. On the basis of these results and those presented elsewhere (16), we propose that a more thorough investigation of the role of the *ERG3* gene as a determinant of azole susceptibility in the clinical setting is warranted. These investigations should not only seek to identify *erg3* null mutants but also consider the effects of variation in *ERG3* transcription, protein expression, and missense mutations on the catalytic activity of Erg3p. Furthermore, the consequences of altered Erg3p activity upon fungal pathogenicity should be simultaneously examined.

## MATERIALS AND METHODS

### Growth and media conditions.

All C. albicans strains were routinely grown on yeast extract-peptone-dextrose (YPD) agar plates or in liquid medium at 30°C and supplemented with uridine (50 µg/ml) when necessary. Transformant selection was carried out on minimal YNB medium (6.75 g/liter yeast nitrogen base without amino acids, 2% dextrose, 2% Bacto agar), supplemented with the appropriate auxotrophic requirements as described for S. cerevisiae (50) or uridine at 50 µg/ml. All C. albicans strains were stored frozen at −80°C in 20% glycerol.

For sterol extraction, strains were grown overnight in YNB, and 100 µl of a solution with 1 × 10^6^ cells/ml were used to inoculate a final volume of 10 ml YNB broth plus 5 µg/ml fluconazole or 0.5% dimethyl sulfoxide (DMSO) (minus fluconazole control) to give a starting concentration of 1 × 10^4^ cells/ml. These cultures were grown at 35°C for 24 h with shaking at 160 rpm. All strains were grown in triplicate.

For RNA extraction, 1 × 10^4^ cells/ml were inoculated in 5 ml YPD and grown at 37°C with shaking for 24 h in the presence of 4 µg/ml of fluconazole or 0.5% DMSO. All strains were grown in triplicate.

### Plasmid construction.

Plasmid pLUX (28), was kindly provided by William Fonzi (Georgetown University). Plasmid pKE1 (51) was previously described. All oligonucleotides used in this study are listed in Table S1 in the supplemental material.

10.1128/mBio.00225-18.9 TABLE S1  Oligonucleotides used in this study. Download TABLE S1, DOCX file, 0.03 MB. Copyright © 2018 Luna-Tapia et al. 2018 Luna-Tapia et al. This content is distributed under the terms of the Creative Commons Attribution 4.0 International license.

The *ERG3* ORF with 5′ and 3′ UTR sequences was amplified from SC5314 genomic DNA (gDNA) with Hi-Fi Platinum *Taq* (Invitrogen) and primer pairs ERG3AMPF-StuI plus ERG3AMPR-SacI or ERG3AMPF2-StuI plus ERG3AMPR-SacI, and cloned between the StuI and SacI restriction sites of pLUX to produce plasmids pLUX-shprERG3 (containing ~582 bp of the *ERG3* promoter) and pLUX-lnprERG3 (containing 1 kb of the *ERG3* promoter), respectively.

Construction of the reporter plasmids for transcriptional analysis, plnERG3prGFPγ and pshERG3prGFPγ, was performed as follows. Different lengths of the *ERG3* promoter were amplified from SC5314 gDNA with primer pairs ERG3lngpF-KpnI plus ERG3prR-SalI (1,000 bp upstream of the start codon) or ERG3shrpF-KpnI plus ERG3prR-SalI (582 bp upstream of the start codon). These promoter fragments were then cloned between the KpnI and SalI sites of pKE1 in place of the *ACT1* promoter. The GFPγ coding sequence was then amplified using primer pair GFPAMPF-SalI plus GFPAMPR-MluI and cloned downstream of the *ERG3* promoter between SalI and MluI sites.

### C. albicans strain construction.

Strains used in this study are listed in Table S2. Gene deletion strains were constructed by the PCR-based approach of Wilson et al. (27), using the *ura3Δ/Δ his1Δ/Δ arg4Δ/Δ* strain BWP17 (kindly provided by Aaron Mitchell, Carnegie Mellon University).

10.1128/mBio.00225-18.10 TABLE S2  Strains used in this study. Download TABLE S2, DOCX file, 0.03 MB. Copyright © 2018 Luna-Tapia et al. 2018 Luna-Tapia et al. This content is distributed under the terms of the Creative Commons Attribution 4.0 International license.

*ERG3* deletion cassettes were amplified by PCR with primer pair ERG3DISF plus ERG3DISR, using pRS-ARG4*Δ*SpeI or pGEM-HIS1 (27) as the template. Each *ERG3* allele was sequentially deleted from strain BWP17 using *ARG4* and *HIS1* markers to generate *erg3Δ/Δ ura3Δ/Δ* gene deletion mutants. Correct integration of deletion cassettes was confirmed at each step by PCR with the following primer sets: ARG4INTF2 plus ERG3AMPR-SacI and ARG4INTR2 plus ERG3AMPF-SacI (*ARG4* integration) or HIS1INTF2 plus ERG3AMPR-SacI and HIS1INTR2 plus ERG3AMPF-SacI (*HIS1* integration). The lack of an intact *ERG3* allele was confirmed using primer pair ERG3DETF and ERG3DETR.

For construction of the C. albicans *upc2*Δ/Δ *ura3*Δ/Δ mutant, each *UPC2* allele was sequentially deleted from strain BWP17 using the *ARG4* and *HIS1* markers. Deletion cassettes were amplified with primer pairs UPC2DISF plus UPC2DISR, using pRS-ARG4*Δ*SpeI or pGEM-HIS1 (27) as the template. Correct integration of deletion cassettes was confirmed by PCR with the following primer sets: UPC2AMPF-KpnI plus ARG4INTR2 and UPC2AMPR-SacI plus ARG4INTF2 (*ARG4* integration), or UPC2AMPF-KpnI plus HIS1INTR2 and UPC2AMPR-SacI plus HIS1INTF2 (*HIS1* integration). The absence of an intact *UPC2* allele was confirmed using primer pair UPC2SEQF1 and UPC2SEQR3.

For the construction of the strains used in the GFPγ reporter assays, plasmids plnERG3prGFPγ and pshERG3prGFPγ were linearized with NheI and transformed into wild-type CAI4 and *upc2Δ/Δ ura3Δ/Δ* strains.

### Stress resistance and hyphal growth assays.

For stress resistance analysis, C. albicans was grown overnight in YPD at 30°C. Cells were washed in sterile deionized water, cell density was adjusted to 10^7^ cells/ml, and 1:5 serial dilutions were performed in a 96-well plate. Cells were then applied to agar plates using a sterile multipronged applicator. Resistance to different stresses was determined on YPD agar containing 8 µg/ml of fluconazole, 0.05% SDS, 1.5 M NaCl, or 500 mM CaCl_2_, with incubation at 30°C during 48 h.

For hyphal growth analysis, 2.5 µl from a cell suspension with 10^7^ cells/ml was spotted on M199 agar or 10% FBS (fetal bovine serum) agar plates, followed by incubation for 96 h at 37°C.

### Quantification of biofilm biomass.

Each C. albicans strain was grown overnight in YPD broth at 37°C with shaking at 200 rpm. Each culture was then washed two times in sterile phosphate-buffered saline (PBS), cell density was adjusted to 1 × 10^6^ per ml in RPMI 1640 medium, and 200 µl was dispensed into each well of a flat-bottomed, 96-well plate (six wells per strain). After incubation at 37°C for 24 h, the plate was rinsed three times with sterile PBS and then stained with 0.01% crystal violet (MP Biomedicals) for 15 min. The plate was again rinsed three times with sterile water, and biofilms were eluted with 95% ethanol (200 µl/well). One hundred twenty-five microliters of resolubilized dye was transferred to a new microtiter plate, and the optical density at 570 nm (OD_570_) was measured using a microplate reader. Four independent experiments were performed.

### Antifungal susceptibility testing.

Antifungal susceptibility testing of all the strains included in this study was performed by using the broth microdilution method described in the CLSI document M27-A3 (30) in a 96-well plate format. All drugs for susceptibility testing used in this study were diluted in DMSO in twofold dilutions at 200 times the final concentration. Fluconazole (Sigma-Aldrich) concentrations tested ranged from 64 µg/ml to 0.0313 µg/ml. RPMI 1640 medium (Sigma-Aldrich) was prepared according to the CLSI document. The medium was buffered with 0.165 M morpholinepropanesulfonic acid (MOPS), and its pH was adjusted using NaOH and HCl. Cell inoculum was ~1 × 10^3^ cells per well. Plates were incubated without shaking at 35°C for 24 or 48 h. The content of each well was carefully resuspended by pipetting up and down before OD_600_ was measured using a Cytation 5 cell imaging multimode reader (Bio-Tek Instruments, Inc.).

### Sterol extraction and quantitation.

Nonsaponifiable lipids were extracted using alcoholic KOH as reported previously (52). Samples were dried in a vacuum centrifuge (Heto) and derivatized by the addition of 100 µl of 90% N,O-bis(trimethylsilyl)trifluoroacetamide (BSTFA)–10% trimethylsilyl (TMS) (Sigma), 200 µl of anhydrous pyridine (Sigma), and heating for 2 h at 80°C. TMS-derivatized sterols were analyzed and identified using gas chromatography-mass spectrometry (GC-MS) (Thermo 1300 GC coupled to a Thermo ISQ mass spectrometer; Thermo Scientific) with reference to retention times and fragmentation spectra for known standards. GC-MS data files were analyzed using Xcalibur software (Thermo Scientific) to determine sterol profiles for all isolates and for integrated peak areas. Percentages of total sterols are given as the mean for three replicates.

### RNA extraction.

Cells were collected by centrifugation at 3,500 rpm for 5 min. Supernatant was poured off, and cells pellets were stored at −80°C. RNA was extracted from the cell pellets using the hot phenol method of Schmitt et al. (53). RNA pellets were eluted in 20 µl of nuclease-free water, and quantity and purity were determined spectrophotometrically by measuring absorbance at 260 nm and 280 nm.

### Quantitative RT-PCR (qRT-PCR).

RNA concentrations were adjusted to 1 µg, and aliquots were treated with DNase I as indicated by the manufacturer (Thermo Scientific). cDNA was synthesized using the Verso cDNA synthesis kit (Thermo Scientific) with random hexamers according to the manufacturer’s instructions. Quantitative PCR was performed with primer pairs that amplify 100 bp of the *ACT1* (ACT1FWDS2 plus ACT1REVS2) or *ERG3* (ERG3qPCR_F2 plus ERG3qPCR_R2) (Table S1) coding sequences using the Maxima SYBR green/ROX quantitative PCR (qPCR) master mix (2×) (Thermo Scientific) as indicated by the manufacturer. Reactions were completed in a 7500 real-time PCR system (Applied Biosystems). Expression levels of *ERG3* were compared to those of *ACT1* (normalizing gene) using the Δ*C*_*T*_ method as described previously (54).

### GFPγ reporter assays.

C. albicans strains expressing GFPγ from the *ERG3* promoter were grown overnight in YPD at 30°C, washed, and subcultured into 10 ml YNB at 1 × 10^6^ cells/ml in the presence of 5 µg/ml fluconazole or 0.5% DMSO, and then incubated at 35°C for 24 h with shaking. Cells were pelleted and transferred to a round-bottomed 96-well plate, and GFPγ fluorescence intensity was quantified using a Cytation 5 cell imaging multimode reader (Bio-Tek Instruments, Inc.) with excitation at 488 nm (9 nm bandwidth) and emission at 507 nm (9 nm bandwidth). Growth was determined by measuring OD_600_ for normalization of the fluorescence signal.

### Ethics statement.

The animals used in this study were housed in American Association for Accreditation of Laboratory Animal Care (AAALAC)-approved facilities at the University of Tennessee Health Science Center (UTHSC). The Institutional Animal Care and Use Committee (IACUC) at UTHSC approved use of all animals and procedures (IACUC protocol numbers 15-081 and 16-156). Mice were given standard rodent chow and water *ad libitum*. Mice were monitored daily for signs of distress, including noticeable weight loss, lethargy, and body condition score. The IACUC at UTHSC uses the Public Health Service (PHS) Policy on Humane Care and Use of Laboratory Animals (55) and the *Guide for the Care and Use of Laboratory Animals* (56) as a basis for establishing and maintaining an institutional program for activities involving animals. To ensure high standards for animal welfare, the IACUC at UTHSC remains compliant with all applicable provisions of the Animal Welfare Act (AWAR) (57), guidance from the Office of Laboratory Animal Welfare (OLAW), and the American Veterinary Medical Association Guidelines on Euthanasia (58).

### Mouse model of vaginal candidiasis.

C57BL/6 mice were administered 0.1 mg of estrogen (β-estradiol 17-valerate; Sigma) dissolved in 0.1 ml sesame oil subcutaneously 72 h prior to inoculation with C. albicans. Estrogen injections were administered weekly thereafter. Stationary-phase cultures of C. albicans isolates were washed three times in sterile, endotoxin-free PBS and resuspended in PBS at 2.5 × 10^8^ cells/ml. Estrogen-treated mice (eight mice in each experimental group) were then inoculated intravaginally with 20 µl of the cell suspension (final inoculum, 5 × 10^6^ cells). Mice were monitored daily for signs of distress, including noticeable weight loss and lethargy. Fluconazole treatment was administered at 50 mg/kg via intraperitoneal injection on days 4 and 7 postinoculation (p.i.). Vaginal lavage was performed on day 7 and 10 p.i., and fungal colonization was quantified as CFU by plating serial dilutions of the lavage fluid onto YPD agar plates containing 50 µg/ml of chloramphenicol. PMN counts within the lavage fluid samples were also enumerated as an indicator of inflammation. Smears of lavage fluid samples were stained by the Papanicolau technique, and the number of PMNs in five nonadjacent fields was counted by using a light microscope with a 40× objective.

### Mouse model of disseminated candidiasis.

C. albicans strains were grown overnight in YPD cultures at 30°C with shaking. Stationary-phase cultures of C. albicans strains were washed twice in sterile, endotoxin-free PBS and resuspended in PBS. BALB/c mice were inoculated via tail vein injection with ~5 × 10^5^ cells in 100 µl of cell suspension. Viable-cell counts of each inoculum were confirmed by plating appropriate dilutions onto YPD agar plates and counting the colonies formed after 48 h.

### Pathogenesis experiment.

Groups of six animals per strain were infected and monitored for 14 days p.i., and those showing signs of distress were humanely sacrificed. Surviving animals by day 14 p.i. were euthanized. The kidneys from each mouse were extracted, weighed, and homogenized in PBS. Serial dilutions of kidney homogenate were plated on YPD agar plates containing 50 µg/ml of chloramphenicol. The number of CFU/gram of kidney tissue was determined from the number of colonies on the plates after 48 h.

### *In vivo* fluconazole treatment experiment.

Groups of eight animals per strain were inoculated via tail vein injection with 100 µl of cell suspension. After 24 h p.i., each group was split into two groups of four animals each. Either group was treated intraperitoneally with 200 µl of 25 mg/kg/day of fluconazole or with vehicle alone (PBS plus 1% DMSO) daily for 6 days (until day 7 p.i.). Mice were monitored daily, and those showing distress were euthanized. Kidneys were removed from each mouse at the time of death, weighed, and homogenized in PBS, and their fungal burdens were determined as described earlier in this article. On day 8 p.i., all surviving animals were sacrificed, their kidneys were removed, and their fungal burdens were determined as explained previously.

10.1128/mBio.00225-18.8 FIG S8  C. albicans *erg3* clinical isolates are phenotypically similar to the shprERG3 strain. The fluconazole susceptibility of the C. albicans SC5314 (wild type), our *erg3*Δ/Δ mutant, the azole-resistant isolate TW17, the previously reported *erg3* clinical isolates CA12 and CA1008, and the so-called *erg3* “leaky” strains CA488 and CA490 was evaluated using the standard CLSI broth microdilution protocol. (A) Following 24-h incubation, growth was measured as OD_600_ and expressed as a percentage of the growth in the no-drug (DMSO alone) control wells of each strain. The means ± standard deviations for three biological replicates are shown. The phenotypes of C. albicans SC5314, CA12, CA1008, CA488, and CA490 were tested under different stresses. (B) Each C. albicans strain was suspended at 1 × 10^7^ cells/ml, serial 1:5 dilutions were prepared in sterile water and applied to agar plates containing fluconazole, SDS, NaCl, or CaCl_2_, using a sterile multipronged applicator. The plates were incubated at 30°C for 48 h and then imaged. (C) Each C. albicans strain was suspended in a solution at 1 × 10^7^ cells/ml, and 2.5 µl was spotted on 10% FBS or M199 agar plates. After incubation at 37°C for 96 h, the resulting colonies were imaged. Download FIG S8, JPG file, 2.8 MB. Copyright © 2018 Luna-Tapia et al. 2018 Luna-Tapia et al. This content is distributed under the terms of the Creative Commons Attribution 4.0 International license.
