# Supplementary figures and images for: Loss of Upc2p-Inducible ERG3 Transcription Is Sufficient To Confer Niche-Specific Azole Resistance without Compromising Candida albicans Pathogenicity
Source: mBio. 2018 May 22;9(3):e00225-18. doi: 10.1128/mBio.00225-18 (PMC5964354; doi:10.1128/mBio.00225-18)

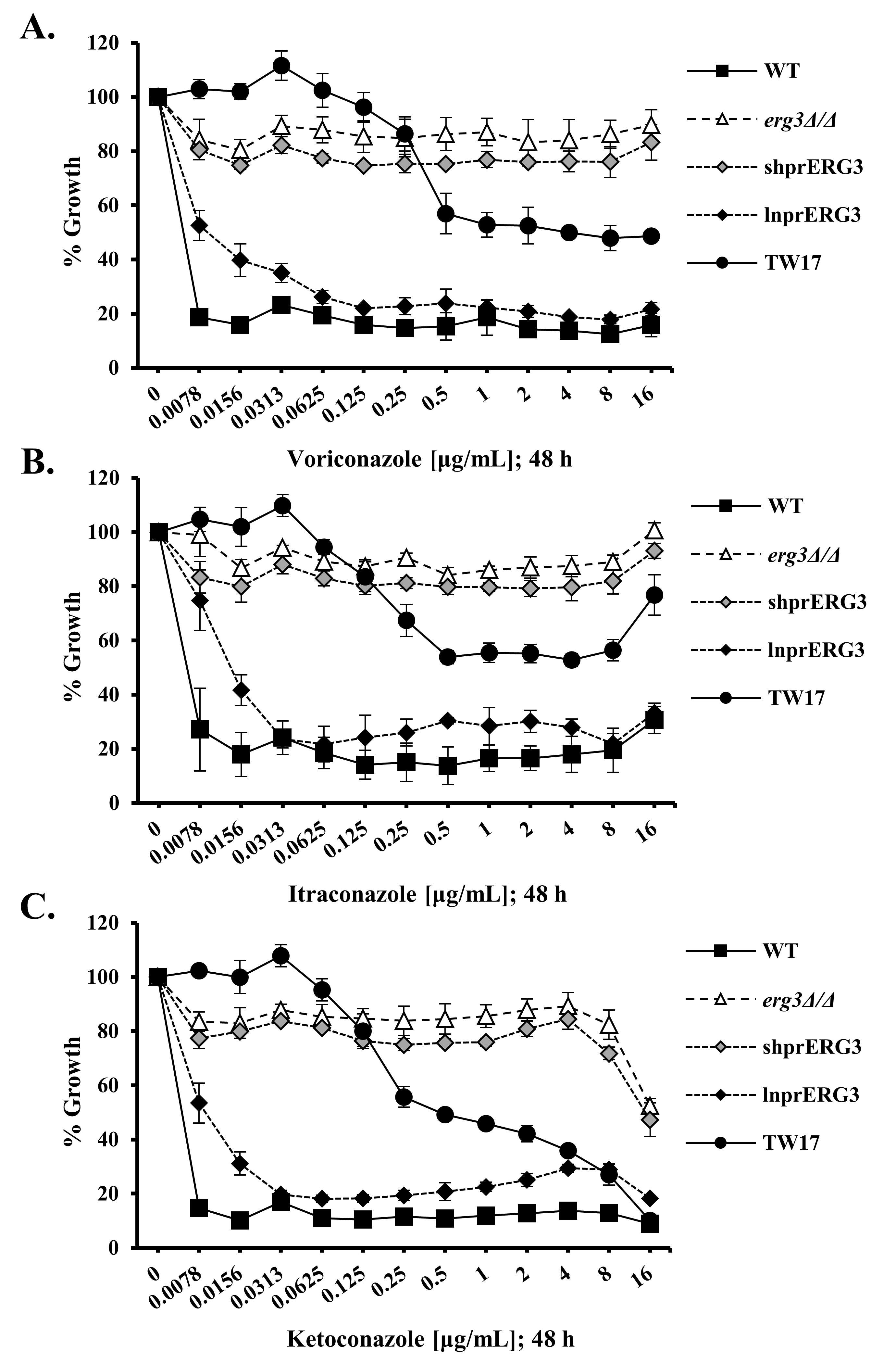

Supplement: FIG S1 [file mbo003183893sf1.jpg]

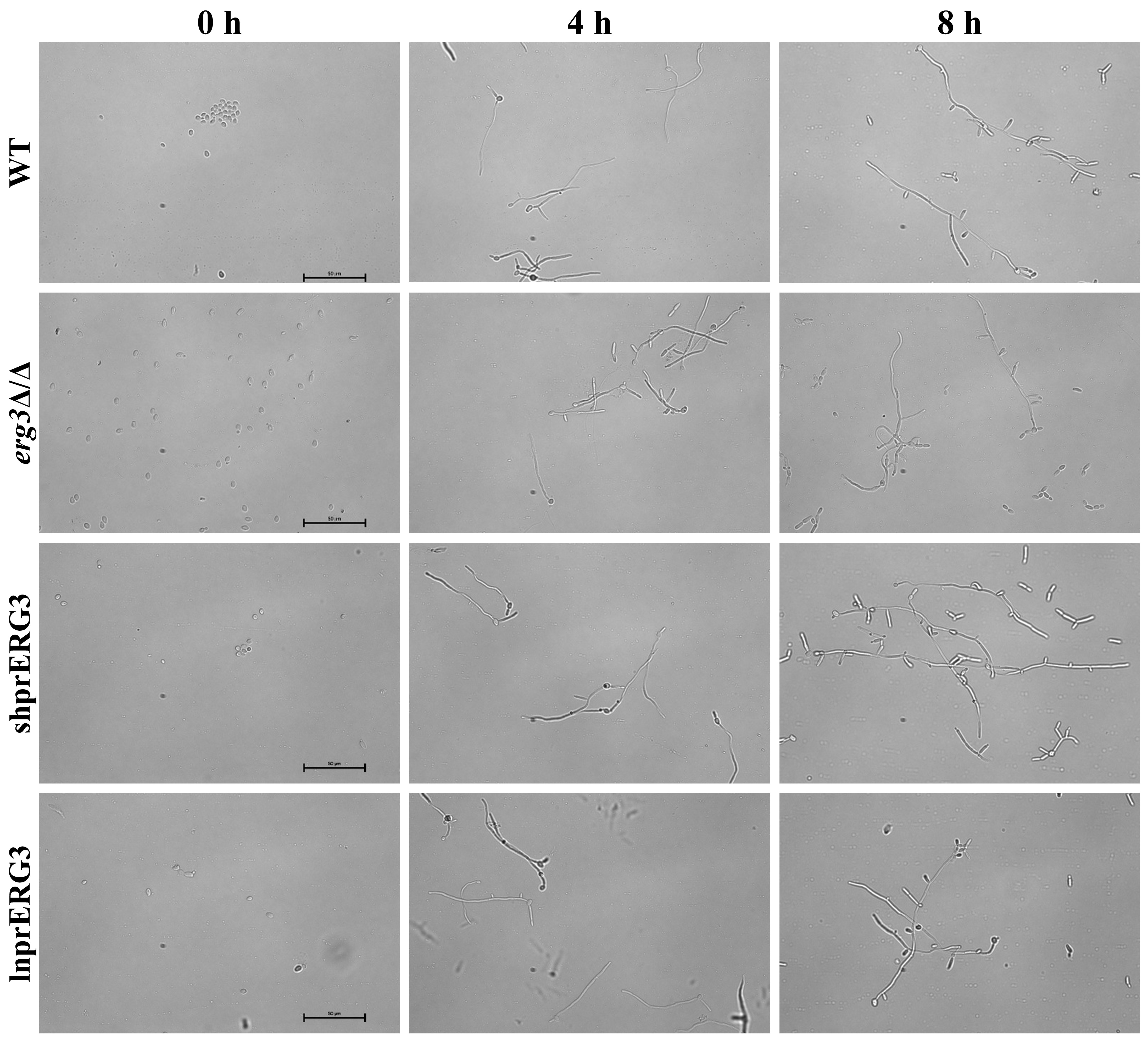

Supplement: FIG S2 [file mbo003183893sf2.jpg]

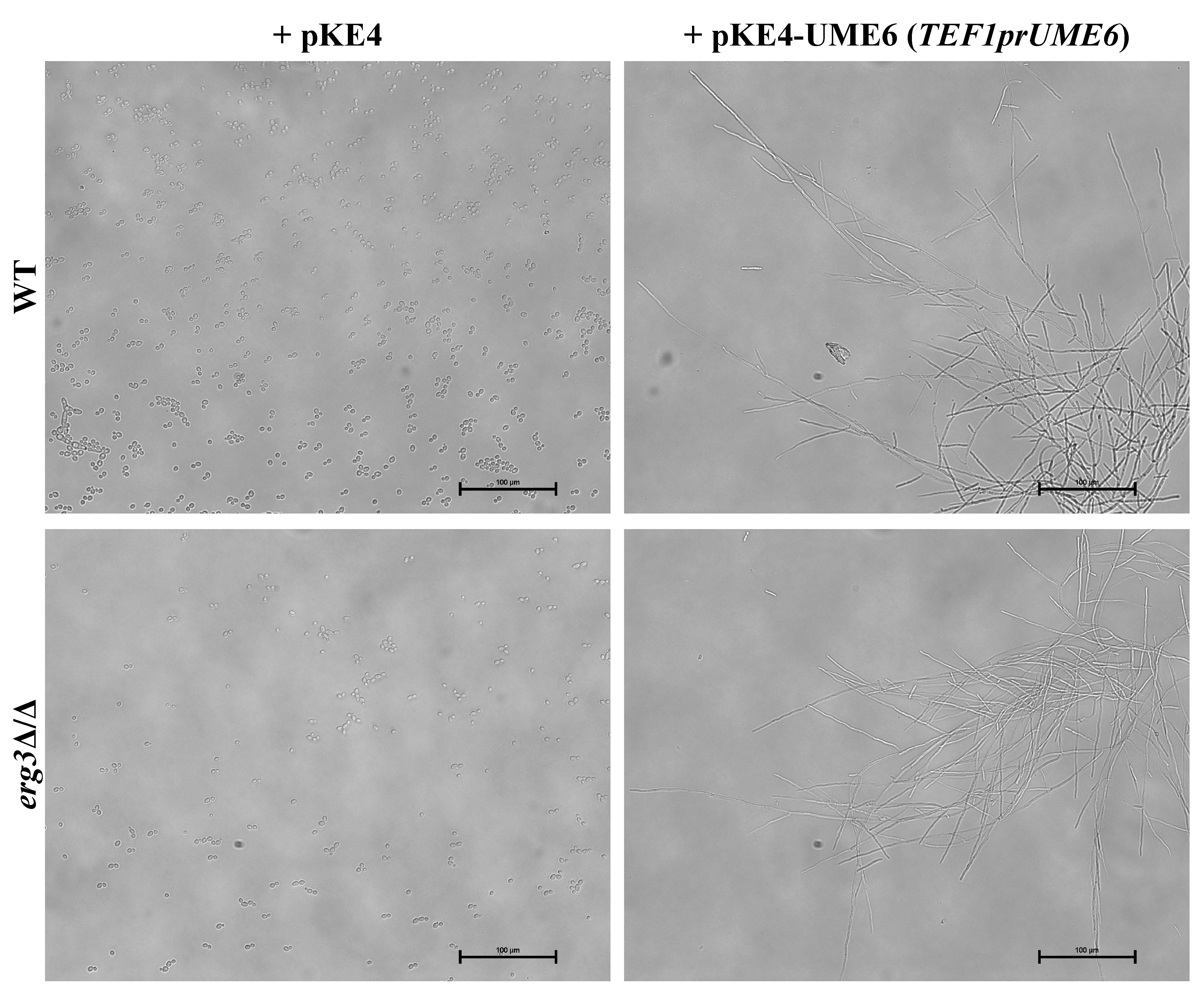

Supplement: FIG S3 [file mbo003183893sf3.jpg]

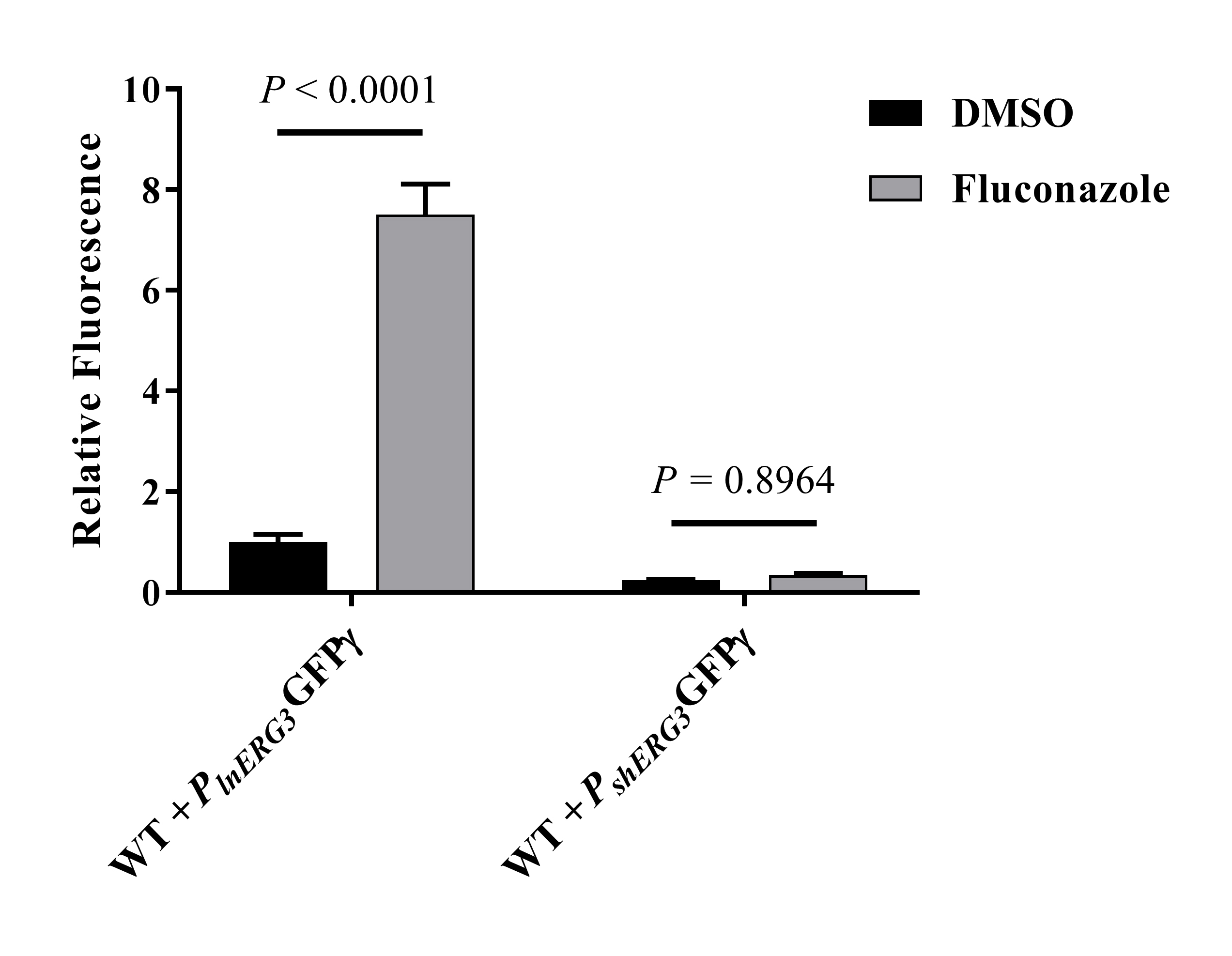

Supplement: FIG S4 [file mbo003183893sf4.jpg]

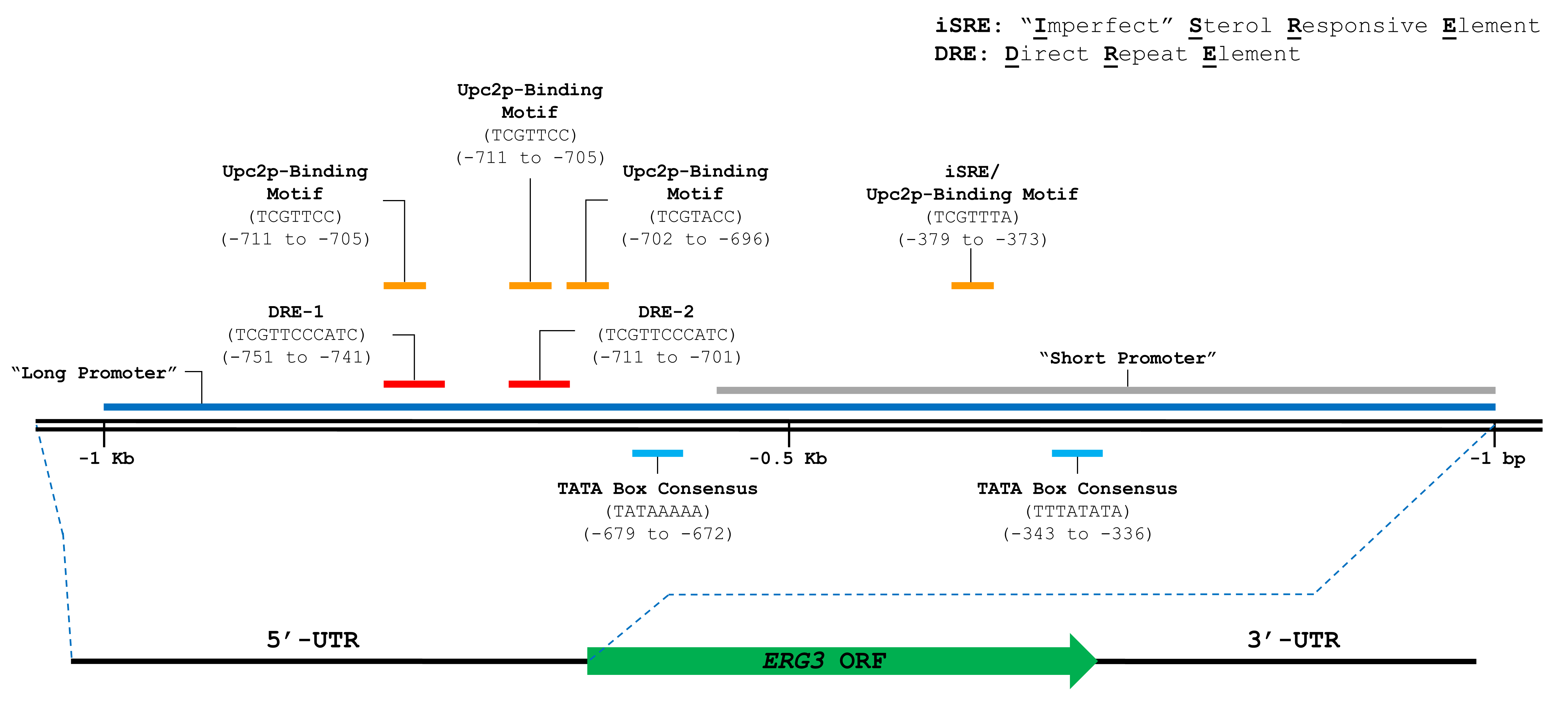

Supplement: FIG S5 [file mbo003183893sf5.jpg]

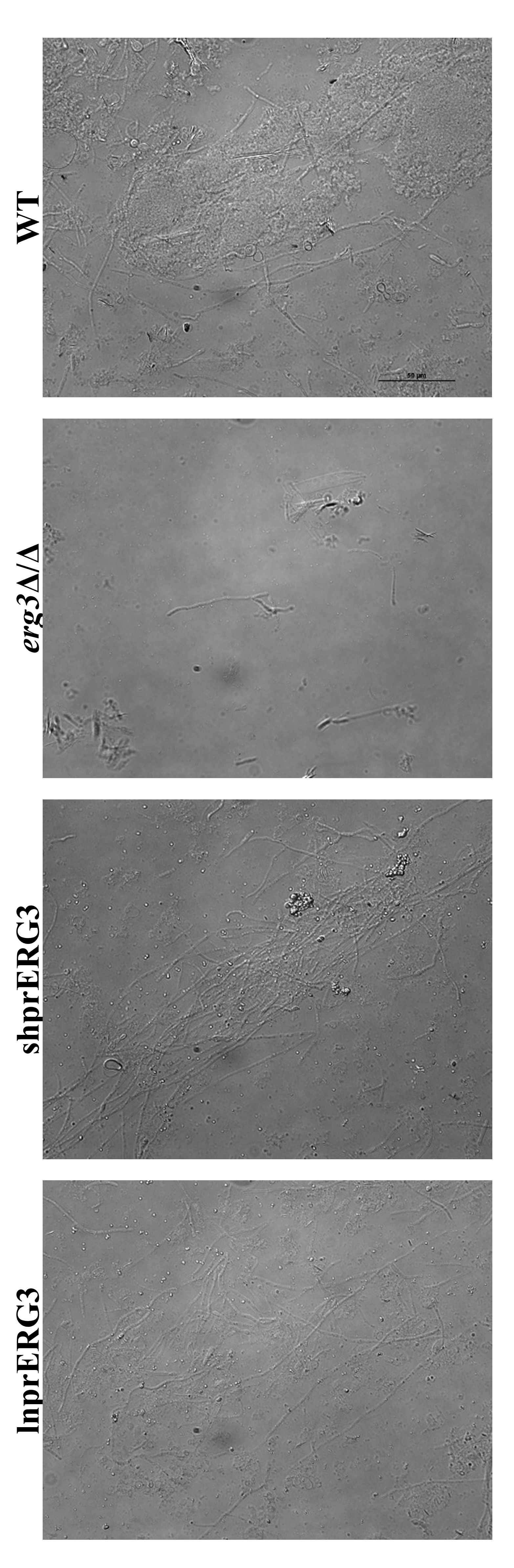

Supplement: FIG S6 [file mbo003183893sf6.jpg]

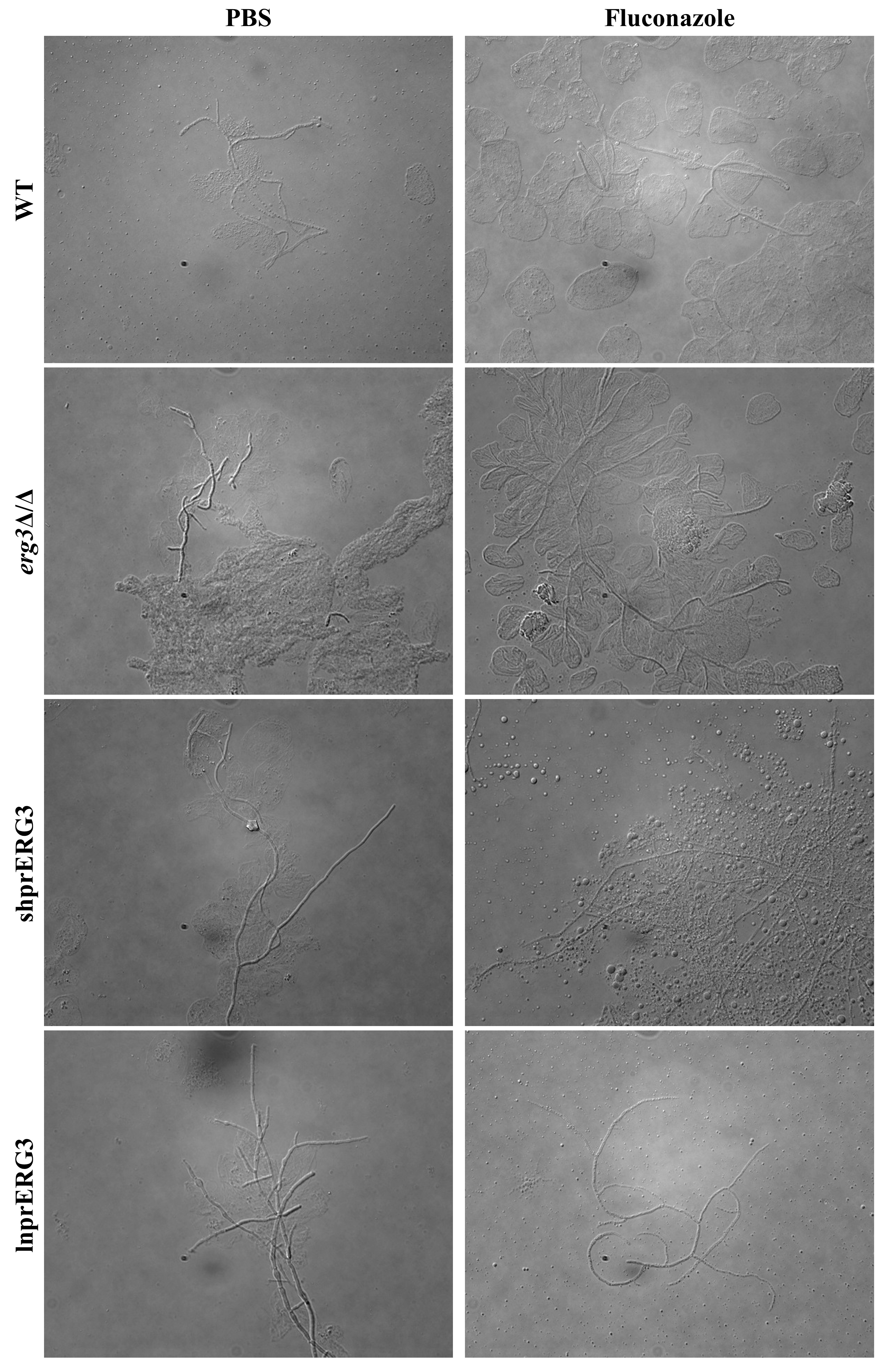

Supplement: FIG S7 [file mbo003183893sf7.jpg]

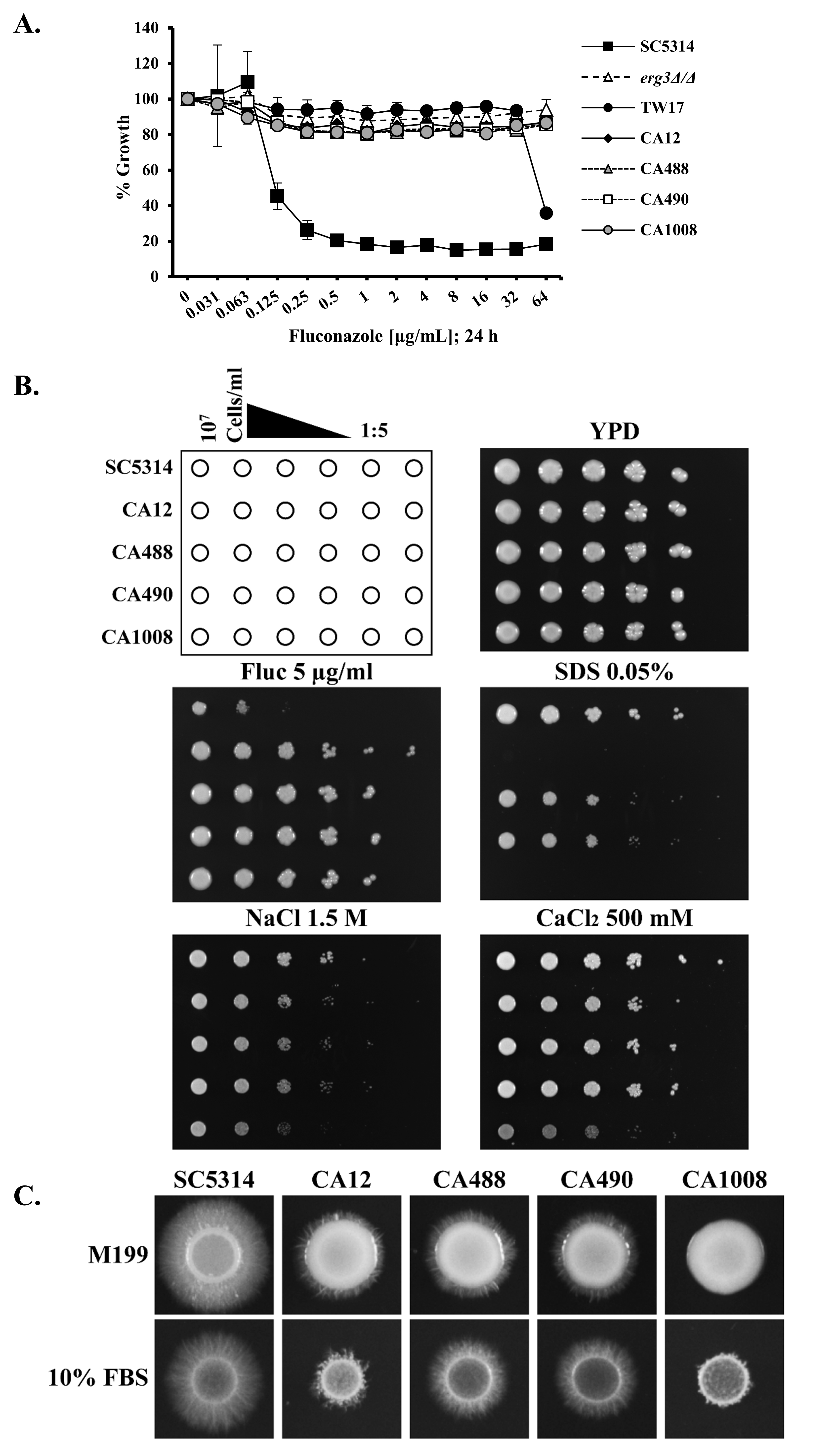

Supplement: FIG S8 [file mbo003183893sf8.jpg]
