# Supplementary material for: Loss of Upc2p-Inducible ERG3 Transcription Is Sufficient To Confer Niche-Specific Azole Resistance without Compromising Candida albicans Pathogenicity
Source: mBio. 2018 May 22;9(3):e00225-18. doi: 10.1128/mBio.00225-18 (PMC5964354; doi:10.1128/mBio.00225-18)
Supplement: TABLE S1 [file mbo003183893st1.docx]

**Table S1. List of oligonucleotides used in this study.**

| **PRIMER NAME** | **SEQUENCE (5’-3’)** |
| --- | --- |
| **ACT1FWDS2** | ACTACCATGTTCCCAGGTATTG |
| **ACT1REVS2** | CCACCAATCCAGACAGAGTATT |
| **ARG4INTF2** | AAGCTAGTGTGGAAAGAAGAG |
| **ARG4INTR2** | AATGACTGAATTATGTCGGTC |
| **ERG3AMPF2-StuI** | TCA**AGGCCT**CAATCTACATAGAGGAGTAGAACGG |
| **ERG3AMPF-StuI** | TCA**AGGCCT**GACTACGCGAGACCACACTTGC |
| **ERG3AMPR-SacI** | TCA**GAGCTC**GTGAAATTACTTACAATATGGAG |
| **ERG3DETF** | GCTGATGTTTTCCCTAAAGATGG |
| **ERG3DETR** | GTAAGAGTTACCAAGTCTATCCC |
| **ERG3DISF** | CCCTTCCCATTTCTTTCCCTATTGTGCATATAAGTTCAATCTTTTTTTCTTTCTTTCGGATTCGGTTTAG*TGTGGAATTGTGAGCGGATA* |
| **ERG3DISR** | GGAAAAATAGTCAATGGTCCAAAACAAAGATGTACCAATCATTGTTCAACATATTCTCTATCGTCAACTT*GTTTTCCCAGTCACGACGTT* |
| **ERG3lngpF-KpnI** | TCA**GGTACC**TCAATCTACATAGAGGAGTAGAACGG |
| **ERG3prR-SalI** | TCA**GTCGAC**GGTAGTAAAATTAGCTAAACCG |
| **ERG3qPCR-F2** | CTGGTCTAATGACCCAGTTGTC |
| **ERG3qPCR-R2** | CCAAGTCTATCCCAAAGAGTAGTG |
| **ERG3shrpF-KpnI** | TCA**GGTACC**GACTACGCGAGACCACACTTGC |
| **GFPAMPF-SalI** | TCA**GTCGAC**ATGTCTAAAGGTGAAGAATTATTC |
| **GFPAMPR-MluI** | TCA**ACGCGT**TTATTTGTACAATTCATCC |
| **HIS1INTF2** | ACTGTATCCTCTTCTGTCCCC |
| **HIS1INTR2** | CGACCATATGGGAGAGCTCCC |
| **LUXINTDETF** | CTGACCTTTAGTCTTTCCTGC |
| **LUXINTDETR** | CAGTAGTACTTGTTGTTGTATCG |
| **UPC2AMPF-KpnI** | TCA**GGTACC**TCCTGCAAAATAAACACGTGCC |
| **UPC2AMPR-SacI** | TCA**GAGCTC**TTACACCACAGTAACGAATCAC |
| **UPC2DISF** | GCAGAGAATCACAGTGAAGTTCTTTGAATCAAGTTCATTGAAGATATAGTTTTCAACCACTATTACTACC*TGTGGAATTGTGAGCGGATA* |
| **UPC2DISR** | AACCCGGCTAGTGTTGTAATAAACCCTACACAGTCGTAAATTCCTATCATCTACGCGGTATTGACCTGTC*GTTTTCCCAGTCACGACGTT* |
| **UPC2SEQF1** | CAATAATGAACCCCCATCAAAG |
| **UPC2SEQR3** | GTCGCCTAAATCTCCCAAGTCG |
| * Engineered restriction enzyme sites are highlighted in bold text and underlined. In italics, regions of homology for amplification of deletion cassettes. | |
