# Supplementary material for: Loss of Upc2p-Inducible ERG3 Transcription Is Sufficient To Confer Niche-Specific Azole Resistance without Compromising Candida albicans Pathogenicity
Source: mBio. 2018 May 22;9(3):e00225-18. doi: 10.1128/mBio.00225-18 (PMC5964354; doi:10.1128/mBio.00225-18)
Supplement: TABLE S2 [file mbo003183893st2.docx]

**Table S2. List of strains used in this study.**

| **Strain** | **Relevant Genotype** | **Reference** |
| --- | --- | --- |
| BWP17 | *ura3∆/∆ his1∆/∆ arg4∆/∆* | ([1](#_ENREF_1)) |
| CAI4 (“Wild-type”) | *ura3∆/∆ HIS1/HIS1 ARG4/ARG4* | ([2](#_ENREF_2)) |
| GP1/GP21 (“Wild-type”) | *ura3∆/∆:URA3 his1∆/∆:HIS1 arg4∆/∆:ARG4* | This study |
| *erg3∆/∆* | *ura3∆/∆:URA3 his1∆/∆ arg4∆/∆ erg3∆:HIS1/erg3∆:ARG4* | This study |
| shprERG3 | *ura3∆/∆:URA3: pr_582bp_ERG3 his1∆/∆ arg4∆/∆ erg3∆:HIS1/erg3∆:ARG4* | This study |
| lnprERG3 | *ura3∆/∆:URA3: pr_1000bp_ERG3 his1∆/∆ arg4∆/∆ erg3∆:HIS1/erg3∆:ARG4* | This study |
| TW17 (Azole^R^) | *ERG3/ERG3* | ([3](#_ENREF_3)) |
| *erg3∆/∆ura3*Δ/Δ | *ura3∆/∆ his1∆/∆ arg4∆/∆ erg3∆:HIS1/erg3∆:ARG4* | This study |
| *upc2*Δ/Δ*ura3*Δ/Δ | *ura3∆/∆ his1∆/∆ arg4∆/∆ upc2∆:HIS1/upc2∆:ARG4* | This study |
| WT *+ P_shERG3_GFPγ* | *ura3∆/∆:URA3: ERG3pr_582bp_:GFPγ HIS1/HIS1 ARG4/ARG4* | This study |
| WT *+ P_lnERG3_GFPγ* | *ura3∆/∆:URA3: ERG3pr_1000bp_:GFPγ HIS1/HIS1 ARG4/ARG4* | This study |
| *upc2*Δ/Δ *+ P_lnERG3_GFPγ* | *ura3∆/∆:URA3: ERG3pr_1000bp_:GFPγ his1∆/∆ arg4∆/∆ upc2∆:HIS1/upc2∆:ARG4* | This study |
| SC5314 | *ERG3/ERG3* | ([4](#_ENREF_4)) |
| CA12 (Azole^R^) | *ERG3^W332R^/ERG3^W332R^* | ([5](#_ENREF_5)) |
| CA488 (Azole^R^) | *ERG3^H243N, T330A, A351V^/ERG3^H243N, T330A, A351V^* | ([5](#_ENREF_5)) |
| CA490 (Azole^R^) | *ERG3^D147G, T330A, A351V^/ERG3^D147G, T330A, A351V^* | ([5](#_ENREF_5)) |
| CA1008 (Azole^R^) | *ERG3^K97E, L193P, V237A, A351V, A353T^/ERG3^K97E, L193P, V237A, A351V, A353T^* | ([5](#_ENREF_5)) |
| Azole^R^: Azole resistant clinical isolate. | | |

**References.**

1. **Wilson RB, Davis D, Mitchell AP.** 1996. Rapid hypothesis testing with *Candida albicans* through gene disruption with short homology regions. J Bacteriol **181:**1868 - 1874.

2. **Fonzi WA, Irwin MY.** 1993. Isogenic strain construction and gene mapping in *Candida albicans*. Genetics **134:**717-728.

3. **White TC.** 1997. Increased mRNA levels of *ERG16*, *CDR*, and *MDR1* correlate with increases in azole resistance in *Candida albicans* isolates from a patient infected with human immunodeficiency virus. Antimicrob Agent Chemother **41:**1482-1487.

4. **Maestrone G, Semar R.** 1968. Establishment and treatment of cutaneous *Candida albicans* infection in the rabbit. Naturwissenschaften **55:**87-88.

5. **Martel CM, Parker JE, Bader O, Weig M, Gross U, Warrilow AG, Rolley N, Kelly DE, Kelly SL.** 2010. Identification and characterization of four azole-resistant *erg3* mutants of *Candida albicans*. Antimicrob Agents Chemother **54:**4527-4533.
